# Genetic diversity and structure of the 4^th^ cycle breeding population of Chinese fir (*Cunninghamia lanceolata* (lamb.) hook)

**DOI:** 10.3389/fpls.2023.1106615

**Published:** 2023-01-27

**Authors:** Yonglian Jing, Liming Bian, Xuefeng Zhang, Benwen Zhao, Renhua Zheng, Shunde Su, Daiquan Ye, Xueyan Zheng, Yousry A. El-Kassaby, Jisen Shi

**Affiliations:** ^1^ State Key Laboratory of Tree Genetics and Breeding, Co-Innovation Center for Sustainable Forestry in Southern China, College of Forestry, Nanjing Forestry University, Nanjing, China; ^2^ Key Laboratory of Timber Forest Breeding and Cultivation for Mountainous Areas in Southern China, Fujian Academy of Forestry Science, Fuzhou, China; ^3^ Department of Tree Improvement, Yangkou State-owned Forest Farm, Nanping, China; ^4^ Department of Forest and Conservation Sciences, Faculty of Forestry, The University of British Columbia, Vancouver, BC, Canada

**Keywords:** Chinese fir, breeding population, RAD-seq, genetic structure, genetic diversity

## Abstract

Studying population genetic structure and diversity is crucial for the marker-assisted selection and breeding of coniferous tree species. In this study, using RAD-seq technology, we developed 343,644 high-quality single nucleotide polymorphism (SNP) markers to resolve the genetic diversity and population genetic structure of 233 Chinese fir selected individuals from the 4^th^ cycle breeding program, representing different breeding generations and provenances. The genetic diversity of the 4^th^ cycle breeding population was high with nucleotide diversity (*P_i_*) of 0.003, and *H_o_* and *H_e_* of 0.215 and 0.233, respectively, indicating that the breeding population has a broad genetic base. The genetic differentiation level between the different breeding generations and different provenances was low (*F_st_* < 0.05), with population structure analysis results dividing the 233 individuals into four subgroups. Each subgroup has a mixed branch with interpenetration and weak population structure, which might be related to breeding rather than provenance, with aggregation from the same source only being in the local branches. Our results provide a reference for further research on the marker-assisted selective breeding of Chinese fir and other coniferous trees.

## 1 Introduction

*Cunninghamia lanceolata* (Lamb.) Hook of the genus *Cunninghamia* in the Cupressacaes family (2n = 22) is a Quaternary ice age relict species and is considered one of the most economically important timber species in southern China. The species is widely distributed in 17 provinces and autonomous regions of China and has rich genetic diversity (Bian et al., 2014). The species has been under cultivation for over 3,000 years and currently covers ~10 million hectares, accounting for 17.3% of the dominant tree species in China’s plantation forests. Genetic improvement activities of Chinese fir started in 1950s, mostly through conventional breeding. At present, the Chinese fir breeding program is in its 4^th^ breeding cycle, which is characterized by the selection and establishment of the 4^th^ cycle breeding population. Phenotypic variation of a multitude of biological traits of Chinese fir is known to be affected by both climate and geography. However, information regarding the neutral variation of molecular markers remains scant (Bian et al., 2014). It is anticipated that the use of molecular markers in the Chinese fir breeding program will help resolve the species genetic structure and diversity across populations and ultimately help in the implementation of marker-assisted selective breeding (Zheng et al., 2015; He et al., 2021).

A species breeding population represents the core material for genetic improvement. It is often used to generate a structured pedigree for genetic evaluation, mainly by implementing a specific mating design among the populations’ members. To prevent genetic variability erosion in the Chinese pine 4^th^ cycle breeding population, rigorous genetic diversity assessment is required. The extent of genetic diversity within a population determines its resilience to unexpected environmental contingencies and successful reproduction and recruitment. Thus, the assessment of genetic diversity and population genetic structure is important for the effective conservation and utilization of coniferous tree populations as well as for the thorough development of their breeding programs (Cai et al., 2020).

Analysis of genetic diversity and population structure of forest tree populations has been mostly based on molecular genetic markers, such as random amplified fragment length polymorphism (RAPD), amplified fragment length polymorphism (AFLP), and simple sequence repeat (SSR) (Chung et al., 2004; Cao et al., 2012; Duan, 2014; Yang et al., 2018). SSR and RAPD markers were used to analyze the genetic diversity of the first three Chinese fir breeding populations (1^st^, 2^nd^, and 3^rd^ cycles) (Li, 2001; Li et al., 2007; Ouyang et al., 2014; Li et al., 2017), and high levels of population genetic diversity were reported. Recently, the use of SNP markers has become common due to their stability, high resolution, wide distribution, and strong differentiation between germplasms (Xia et al., 2019; Zheng et al., 2019). Using specific-locus amplified fragment sequencing (SLAF-seq) technique, Zheng et al. (2019) developed a genome-wide SNP panel for 221 Chinese fir clones. However, *Picea abies* was used as the reference genome for the SNP selection.

High-throughput sequencing technologies generate substantial high-density SNP information, thereby offering opportunities for the development of new strategies for population genetics research. Among them, simplified genome sequencing technologies are widely used as they are free from the “reference genome” constraints. These include restriction-site related DNA sequencing (RAD-seq) (Bus et al., 2012), 2b-RAD sequencing based on RAD-seq (Wang et al., 2012), polymorphic sequence sequencing with reduced complexity (CRoPS) (Altshuler et al., 2000), specific-locus amplified fragment sequencing (SLAF-seq) (Sun et al., 2013), genotyping-by-sequencing (GBS) (Elshire et al., 2011), and reduced representation libraries sequencing (RRLS) (Van Tassell et al., 2008). RAD-seq technology has proven to be an effective sequencing technology for obtaining genome-wide genomic information at low costs and has been extensively used without dependance on “reference genome” (Miller et al., 2007; Zhou et al., 2018; Brandrud et al., 2019). RAD-seq simplified sequencing technology is widely used for plant and animal marker development, population structure analysis, and high-density genetic mapping (Emerson et al., 2010; Catchen et al., 2011; Lexer et al., 2014; Lozier, 2014; Zhou et al., 2016). Although RAD-seq technology is promising, it has not been widely used yet in the population genetic diversity and genetic structure analyses of Chinese fir.

Here, we used the Chinese fir 4^th^ cycle breeding population as the study material to develop high-quality SNPs markers based on RAD-seq simplified genome technology. We expect that this development will not only help elucidating the genetic structure and diversity of the Chinese fir advanced generation breeding population, but also provide theoretical basis and reference for the development and establishment of breeding population and parental selection of seed orchards.

## 2 Materials and methods

### 2.1 Plant material

**Figure 1:**
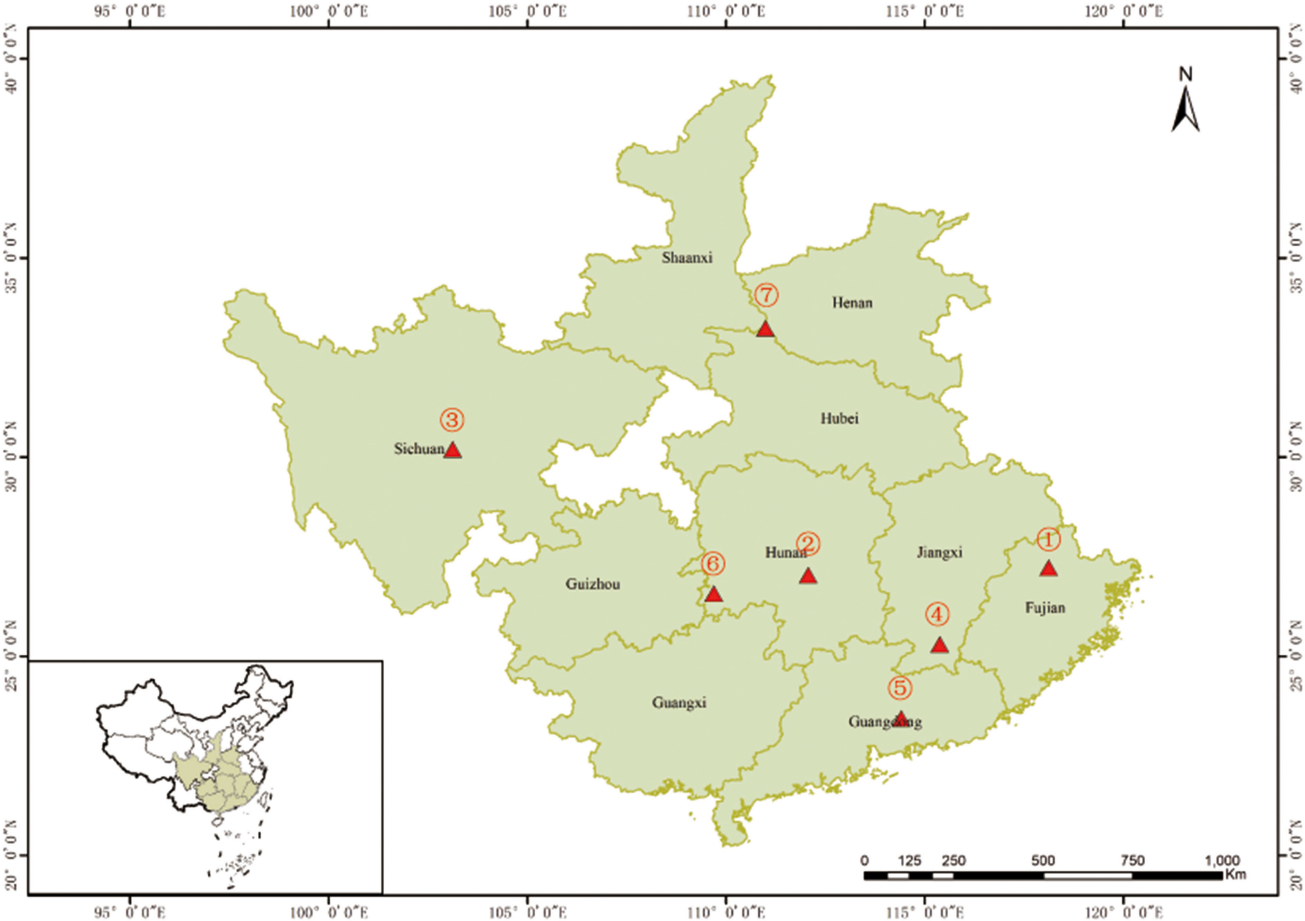
Origin of the 233 Chinese fir germplasm. ①~⑦ represent the seven Chinese fir provenances (Fujian, Hunan, Sichuan, Jiangxi, Guangdong, the boundary of Hunan, Guizhou and Guangxi, and the boundary of Shaanxi, Henan and Hubei). The mixed sources are not shown in the figure.

The Chinese fir 4^th^ cycle genetic improvement population initially was selected for fast growth, high wood quality, and disease resistance. Individuals in this population were selected over three cycles of intensive genetic evaluation and were benchmarked against natural stands’ seedstock. The population is comprised of 233 individuals selected for the above-mentioned attributes along with the added knowledge of their flowering propensity (data generated from three years observations post grafting), which according to the genealogical records could be divided into four generations: 1^st^ (n=43), 2^nd^ (n=141), 3^rd^ (n=38), and 4^th^ (n=11) (**Supplementary Table 1**), thus covering eight geographical Chinese fir origins (**Figure 1**).

### 2.2 DNA extraction and sequencing

Current year fresh needles were collected from 233 Chinese fir trees of the 4^th^ cycle selection population growing in the Yangkou State-owned Forest Farm (Fujian Province) and then preserved on dry ice. DNA was extracted using the Tiangen Biotech kit (DP-320-02), and its quality was checked using Qubit (Thermo Fisher Scientific, Waltham, MA) and Nanodrop (Thermo Fisher Scientific, Waltham, MA) with TE buffer as the blank. DNA purity and integrity were checked using 1% polyacrylamide gel electrophoresis.

Sequencing libraries of the 233 Chinese fir germplasm were constructed using the RAD-seq simplified gene sequencing technology. The quality-checked genomic DNA was enzymatically digested with EcoRI, and samples were double-end sequenced on the Illumina HiSeq 2500 platform to an average depth of 10×. The reference genome was assembled and spliced from the 233 genotypes using the simplified genome sequencing data assembled using the Stacks (Version 1.46) software (Catchen et al., 2011) and its sequencing was also completed by the Stacks (Version 1.46) software. Raw sequencing data containing splice information, low-quality bases, and other information that interferes with downstream analysis were removed to ensure proper data analysis. The FASTP (Version 0.18.0) software (Chen et al., 2018) was used for filtering with the following criteria: 1) removal of sequences lacking the EcoRI restriction sites; 2) removal of low-quality reads (the number of bases with quality Q ≤ 20, which accounted for over 50% of the entire read); 3) elimination of reads containing adapter information; and 4) exclusion of reads with N ratio > 10%.

### 2.3 High*-*quality SNP marker development

The BWA-MEM method of the Burrows-Wheeler-Aligner (v0.7.16a-r1181) software (Li and Durbin, 2009) was used to compare the high-quality reads of each sample with the assembled population tags, with the variant detection software GATK (McKenna et al., 2010) being used for population SNP detection. Using the Plink software (Purcell et al., 2007), the initial SNPs were screened based on the following criteria: 1) indels were removed; 2) only double alleles were retained; 3) Hardy-Weinberg equilibrium (HWE) was met; 4) linkage disequilibrium (LD) between loci was < 0.2; 5) to compare the differences in genetic diversity parameters under different filtering criteria, three sets of criteria were set: ①*MAF*>0.01, Call rate>0.9, ②*MAF*>0.05, Call rate>0.8 and ③*MAF*>0.05, Call rate>0.9. Finally, high-quality SNPs were obtained for genetic diversity analysis.

### 2.4 Data analysis

Using Plink software (Purcell et al., 2007), genetic diversity parameters, including observed (*H_o_*) and expected heterozygosity (*H_e_*), and inbreeding coefficient (*F*) were measured. To equalize the sample size of each population, clusters with larger sample sizes (e.g., G2, FJ) were randomly sampled each time with repeated sampling to calculate the genetic diversity parameters, and finally the mean was estimated. Using the Vcftools software (https://vcftools.github.io/man_latest.html) to 1) calculate nucleotide diversity parameters (*P_i_*); 2) number of conversions and reversals (i.e., structural variation); and 3) calculate Ts/Tv values. The R package StAMPP’s stamppAmova (https://rdrr.io/cran/StAMPP/man/stamppAmova.html) and PopGenome (https://cran.r-project.org/web/packages/PopGenome/index.html) were used for the analysis of molecular variance (AMOVA) and also for estimating the genetic differentiation indices both between the four generations and between the different geographical origins. The SNP data were used to construct a phylogenetic tree for the 4^th^ cycle breeding population using the MEGA 6 software (Tamura et al., 2013). Using the neighbor-joining method with bootstrap values set to 1,000, the phylogenetic tree was constructed using the Kimura 2-parameter model. The ped format file was first exported by Plink software, and then the Admixture (Version 1.3.0) software (Alexander et al., 2009) was used to calculate the Q values and the final population structure was determined. This assumed that the number of sampled sub-groups (K) ranged between 1 and 9, and the valley value of the cross-validation error rate was used as the optimal number of bins. A Q value > 0.6 indicated a single source and pure genetic background, while a Q value < 0.6 indicated a mixed source and complex genetic background. The software EIGENSOFT’s smartpca (https://www.hsph.harvard.edu/alkes-price/software/) module was used for principal component analysis (PCA). The above graphs were visualized using the R software.

## 3 Results

### 3.1 High quality SNP marker development

After RAD-seq sequencing, as shown in **Supplementary Tables 2** and **3**, we obtained 3,145.8 Gb data from the 233 individuals, with data volumes of 9.9–19.6 Gb for each sample, an average of 13.5 Gb per sample, and the average depth of high quality SNP marker sequencing in each sample was 5.5× (**Supplementary Table 4**), and average read length of 146 bp. After quality control, we retained a total of 3,075.3 Gb, with a 97.8% efficiency rate, with data volumes of 9.4–19.4 Gb per sample, and average of 13.2 Gb per sample. The overall sequencing quality was high (Q20 ≥ 97.27%, Q30 ≥ 92.15%), and the GC content was stable (36.61–37.71%, with average of 37.10%), which met the requirements of subsequent analyses. After removing the overlap, there were 2,188,278 contigs. The total length of the assembled reference genome sequence was 1.11 Gb, with average length of 509 bp and a maximum length of 2,211 bp; N50 length of 539 bp and N90 of 406 bp; and 37.01% GC content. The reference genome was compared with the *Picea abies* genome (http://congenie.org/), and it showed 80.48% match, with the RAD-seq sequencing accuracy being reliable for downstream analysis.

After quality control of the raw data, we detected a total of 27,283,139 SNP markers in the whole population as compared to the reference genome, with an average of one SNP locus per 46 bp. The content and distribution density of different types of SNP variants varied across the genome. Among them, conversion accounted for 60.41%, A/G and C/T accounted for 30.41 and 30% respectively; reversal accounted for 39.59% (A/C, C/G, A/T, and G/T), with C/G accounting for 5.51%. After further filtering, we retained a total of 343,644 (1.26%) high quality SNP markers for subsequent analyses. By comparing the genetic diversity parameters of Chinese fir under the three sets of criteria, the results showed that the parameter values under the first set of criteria were significantly smaller than the other two groups, while the values of various genetic parameters calculated under the third set of criteria were higher than the other two groups. Therefore, the SNP markers filtered by the third set of criteria (i.e., *MAF* > 0.05, Call rate > 0.9) were used as high-quality SNP markers, and 343,644 SNP (1.26%) markers were finally retained for subsequent analyses.

### 3.2 Population genetic diversity

We used the 343,644 high-quality SNP markers to calculate the genetic diversity parameters of the breeding parents of different generations and their origins in the 4^th^ Chinese fir cycle breeding population (**Table 1**). *H_o_* varied between 0.203 and 0.218 (mean of 0.211), while *H_e_* varied from 0.214 to 0.231 (mean of 0.225). Both *H_o_* and *H_e_* were the highest in G2, with *H_o_* at all SNP loci being smaller than *H_e_*, thereby indicating that heterozygous deletions may exist in this Chinese fir germplasm population. G4 had the highest *P_i_* (0.003), which may be related to its inclusion of more provenances, followed by G2, which was similar to G3 and G1. Among the origins, *H_o_* was smaller than *H_e_* in FJ and HN, while *H_e_* was larger than *H_o_* in the remaining provenances, and *P_i_* values are also higher in the other provenances compared to the HN and FJ, probably due to the small sample size (only 3 to 5) in the other provenances, thus suggesting that genetic diversity in each provenance is somewhat related to the population size. This shows that the genetic diversity level of the 4^th^ cycle breeding population was high and had abundant genetic variation.

**Table 1:** Genetic diversity parameters of the Chinese fir 4^th^ cycle breeding population.

Groups	Number	*H_o_*	*H_e_*	*P_i_*	*F*
G1	43	0.211	0.228	0.003	0.067
G2	141	0.217	0.231	0.003	0.055
G3	38	0.213	0.229	0.003	0.0665
G4	11	0.203	0.214	0.003	0.047
mean	58.25	0.211	0.225	0.003	0.058
FJ	182	0.216	0.225	0.003	0.038
HN	18	0.208	0.224	0.003	0.064
SC	3	0.211	0.187	0.005	-0.091
JX	3	0.202	0.184	0.005	-0.07
GD	1	–	–	–	–
XQG	5	0.210	0.205	0.004	-0.021
SYE	1	–	–	–	–
MO	20	0.216	0.226	0.003	0.042
All	233	0.214	0.233	0.003	0.075

G1~G4 denote the 1^st^, 2^nd^, 3^rd^, 4^th^ generation breeding parents, respectively, hereinafter, FJ, HN, SC, JX, GD, XQG, SYE, and MO denote Fujian, Hunan, Sichuan, Jiangxi, Guangdong, Hunan-Guizhou-Guangxi border, Shaanxi-Henan-Hubei border, and mixed provenance, respectively. GD and SYE were not calculated due to small sample size. *H_o_* and *H_e_* denote observed and expected heterozygosity, respectively, *P_i_* denotes nucleotide diversity, and *F* denotes inbreeding coefficient.

### 3.3 Populations genetic differentiation

We assessed the genetic differentiation for different breeding generations and different germplasm sources (**Tables 2**, **3**). Generally, the genetic differentiation level is low (*F_st_* < 0.05), indicating that there was no significant genetic differentiation in Chinese fir between the different provinces and between the breeding populations of the four generations. In contrast, the degree of differentiation between SC, JX and GD was higher. The genetic differentiation among the different breeding generations showed the highest differentiation between G4 and G1, which shared similarity with the nucleotide diversity results, whereas the lowest genetic differentiation was between G2 and G3.

**Table 2:** Genetic differentiation among the different provenances of Chinese fir.

Groups	FJ	HN	SC	JX	GD	XQG
FJ	–					
HN	0.0038	–				
SC	0.0117	0.0136	–			
JX	0.0148	0.0178	0.0221	–		
GD	0.0132	0.0113	0.0211	0.0275	–	
XQG	0.0062	0.0086	0.0135	0.0222	0.0130	–
SYE	-0.0176	-0.0189	-0.0124	-0.0007	-0.0070	-0.0201

FJ, HN, SC, JX, GD, XQG, SYE denote Fujian, Hunan, Sichuan, Jiangxi, Guangdong, Hunan-Guizhou-Guangxi boundary, and Shaanxi-Henan-Hubei boundary provenance, respectively.

**Table 3:** Genetic differentiation among the Chinese fir different breeding population generations.

Generation	G4	G3	G2	G1
4^th^ generation (G4)	–			
3^rd^ generation(G3)	0.0094	–		
2^nd^ generation (G2)	0.0102	0.0032	–	
1^st^ generation (G1)	0.0130	0.0090	0.0091	–

The AMOVA results showed that only 1.29% and 3.02% of the variation originated between breeding population generations and between the different germplasm origins, respectively, and over 96% of the variation was due to among different genotypes (**Table 4**).

**Table 4:** Molecular analysis of variance for the Chinese fir different breeding population generations and different germplasm source locations.

Source of variation	SSD	MSD	df	Variance components	% Variance
Among populations	0.5057	0.1686	3.0000	0.0021	1.29%
Within individuals	17.0844	0.0746	229.0000	0.0746	98.70%
Totals	17.5901	0.0758	232.0000		100%
Among populations	0.6100	0.0871	7.0000	0.0009	3.02%
Within individuals	16.9801	0.0755	225.0000	0.0755	96.98%
Totals	17.5901	0.0758	232.0000		100%

AMOVA analysis among different generations of breeding parents (upper)and AMOVA analysis among different provenances (lower).

### 3.4 Population genetic structure

**Figure 2:**
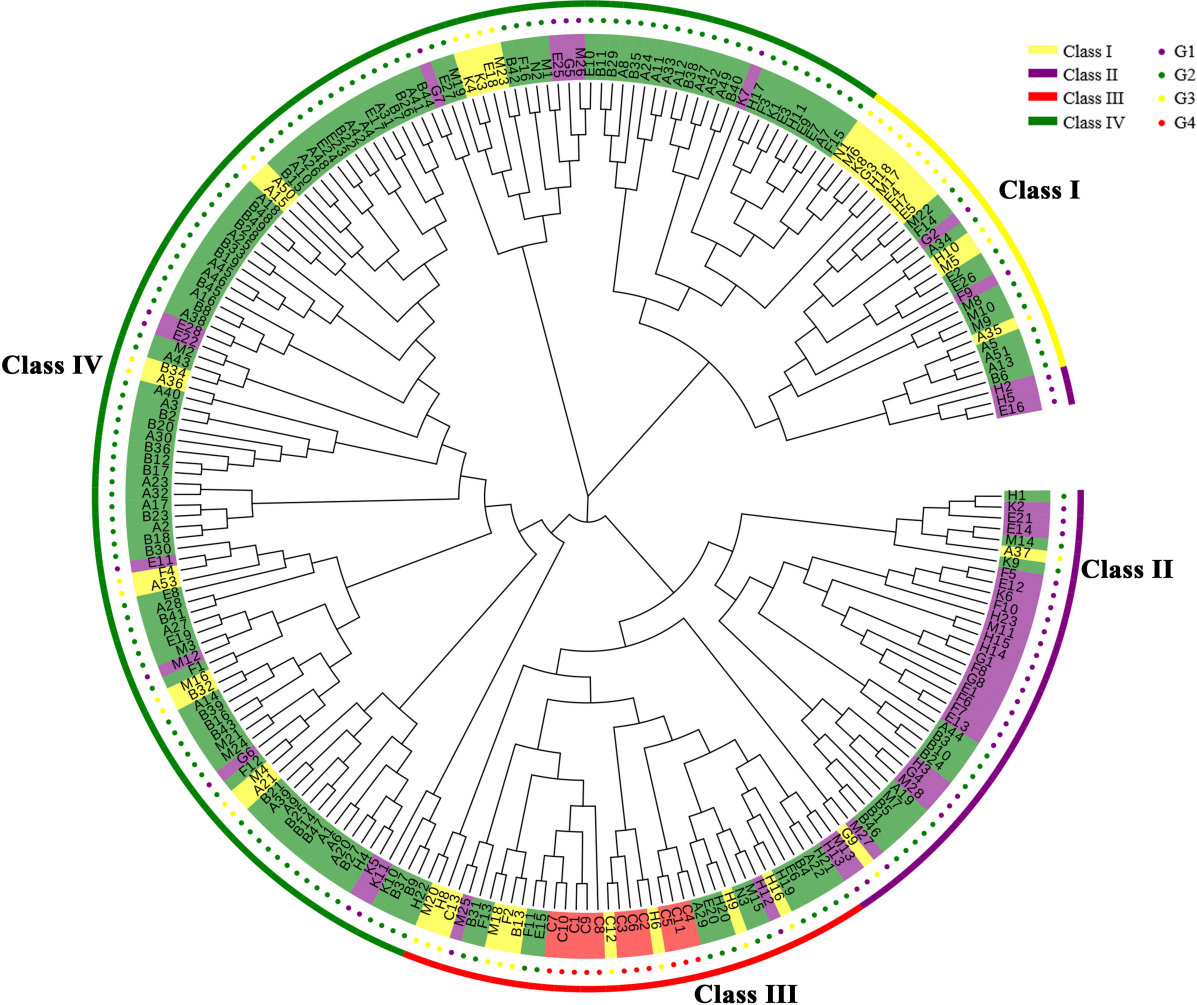
The Chinese fir germplasm phylogenetic tree. The outermost circle in yellow indicates Class I, purple indicates Class II, red indicates Class III, and green indicates Class IV; the inner circle in red indicates G4, yellow indicates G3, green indicates G2 and purple indicates G1.

The 233 Chinese fir individuals of the 4^th^ cycle breeding population can be divided into four differential classes (I-IV) (**Figure 2**). There is large genetic variation among the four classes indicating mixed groups containing individual parents from 3 to 4 generations. Class I contained a minimum of 26 individuals [representing G3 (n=12); G2 (n=12), and G1 (n=2)]; Class II harboured a total of 41 individuals [representing G3 (n=2); G2 (n=12), and G1 (n=27)]; Class III contained 37 individuals [representing G3 (n=10), G2 (n=14), G1 (n=2), and all of G4]; while Class IV contained a maximum of 129 individuals accounting for 55.37% of the tested material [which is dominated by G2 (n=103) and a few G3 (n=14) and G1 (n=12)]. Fifteen individuals of G1 (including F5, E12, and K6) are located in the Class II subclade, confirming the close kinship of these 15 individuals at the molecular level. The evolutionary tree clustered according to provenance (**Supplementary Figure 1**), we found that most provenances were clustered into one group only in the local branches, e.g., most FJ provenances were clustered together, probably due to the larger sample size of the FJ provenance. Therefore, the phylogenetic tree showed that most 4^th^ cycle breeding population clones were mixed to varying degrees, with few outlier samples and no obvious relationship between the division and provenances of the populations, which was probably related to the breeding generations, such that Class IV contained 73.05% of G2 and 36.84% of G3; Class II contained 62.79% of G1; while all of G4 was distributed in Class III.

**Figure 3:**
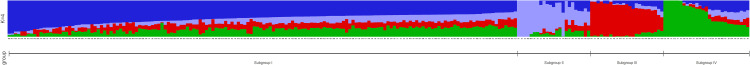
Population structure of 233 Chinese fir germplasm k = 4.

We used the admixture software to calculate the Q values of each sample (**Supplementary Table 5**) and then we grouped the 233 Chinese fir individuals (**Figure 3**, **Supplementary Figures 2-4**). Based on the valley of the cross-validation error rate, we determined that the optimum number of subgroups to be four, thereby indicating that these Chinese fir trees may have come from four original ancestral sources, with the four subgroups (I-IV) containing 160, 23, 23, and 27 individuals, respectively. Subpopulation I had the most complex genetic background, with 40 individuals with Q > 0.6 and 75% of the material having a poorly defined genetic composition. This suggested that there may have been a genetic exchange between these individuals, indicating that the parents may have been used multiple times for crossing in the ongoing breeding process. All of G4 and 75% of G2 comprised subpopulation I. Subpopulation III had greater genetic background purity, which was dominated by G2, where 21 individuals have a Q value > 0.6, probably associated with most samples from G2. Subpopulation II contained G3 (n=8), G2 (n=11), and G1 (n=4), of which 15 individuals have Q values > 0.6. Additionally, 74% of the material in subpopulation IV was from G1, with the remainder from G2, and 14 individuals having Q values > 0.6. All four subgroups retained a proportion of the same genetic material, thus facilitating gene exchange, resulting in a similar genetic background of the breeding parents from different origins. However, the genetic background of the breeding parents from different germplasm sources was similar. Although the subpopulation divisions do not match the provenance of the test material, it only showed some local correlation, thus suggesting that the Chinese fir germplasms may have mixed ancestry or gene flow, which matches the phylogenetic tree results.

**Figure 4:**
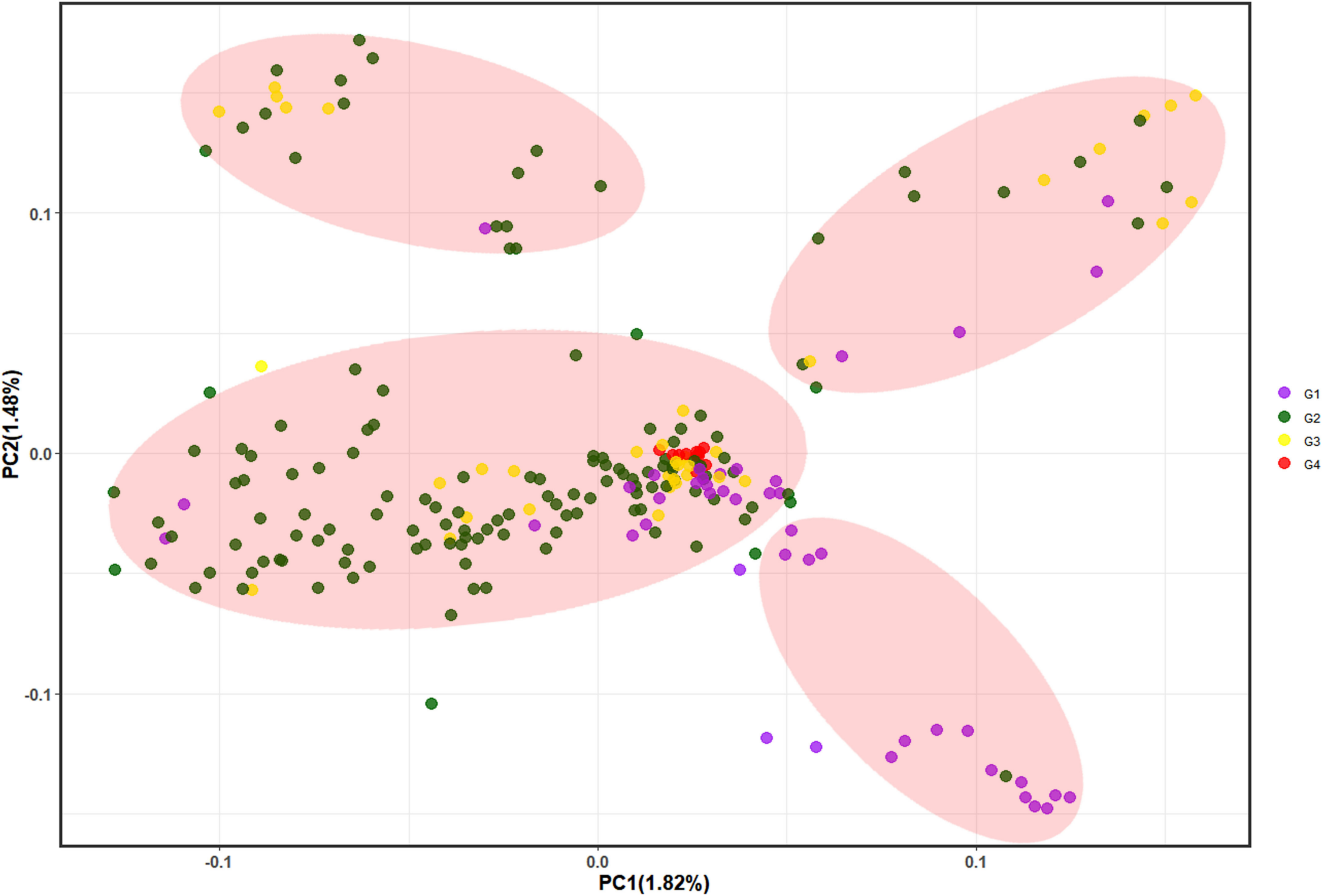
Principal component analysis where PC1 and PC2 represent the first and second principal components, respectively. G1~G4 represent the 1^st^, 2^nd^, 3^rd^ and 4^th^ generation breeding parents, respectively.

PCA showed that the first 10 principal components explained only 11.29% of the variance, with each principal component explaining < 2%, thus indicating that only few SNPs could delineate the subgroups and discriminate between individuals. We selected the first three principal components (PC1 = 1.82%, PC2 = 1.48%, and PC3 = 1.45%) and plotted them in pairs (**Figure 4**, **Supplementary Figure 5**), which divided the 233 individuals into four groups. These results showed that G4 is relatively concentrated in the middle cluster, thus reflecting the close genetic distance between samples within G4. Most G2 and G3 were clustered together, while G1 was more dispersed. Furthermore, elucidating the Chinese fir population genetic structure (maybe related to the breeding generations which unintentionally mixed their genetic background) showed that it does not correspond to the provenance. The studied germplasm indicated that parents from different origins (provenances) were more dispersed, while those from the same provenance were clustered together. In summary, there was overlap and crossover between the four groups and a high degree of admixture between groups, thereby indicating different degrees of interpenetration between groups, which was consistent with both the phylogenetic tree and population genetic structure analysis results.

## 4 Discussion

### 4.1 Reliability of RAD*-*seq for simplified sequencing

With the release of the first version of the *Populus trichocarpa* genome (Tuskan et al., 2006), the era of forest tree genomes had officially started, with the genome-wide information of several tree species being published. However, genomic research progress in coniferous trees is still slow as compared to other plants due to the technical difficulties caused by their very large genomes, high sequencing costs, and gene structure annotation. To date, only a few coniferous tree species genomes have been released (e.g., *Picea abies* (Nystedt et al., 2013), *Pinus taeda* (Zimin et al., 2017), *Pinus lambertiana* (Stevens et al., 2016), *Pseudotsuga menziesii* (Neale et al., 2017), *Pinus tabuliformis* (Niu et al., 2022)). This undoubtedly led to the rapid development of genomic information of these species at the molecular level. Although whole genome of the Chinese fir has not yet been published, very limited genome-level studies are available. In this study, we attempted to construct a reference genome of Chinese fir using RAD-seq simplified sequencing technology for the species 4^th^ cycle breeding population, and obtained a 1.11 Gb-sized genome with a 37.01% GC content, higher than the 36.04% estimated by K-mer analysis (Lin et al., 2020). This estimate is similar to that of *Picea abies* (37.90%) (Nystedt et al., 2013) and *Pinus massoniana* (37.95%) (Bai et al., 2019), and was lower than that of *Cryptomeria japonica* (48.00%) (Nagano et al., 2020), probably due to the lower sequencing depth and lower coverage of the simplified genome sequencing in this study.

The RAD-seq simplified sequencing technique is developed to generate a wider range of SNP markers. It is a cost-effective genotyping technique that detects variant information on a genome-wide scale, but the quality of the obtained SNPs is usually variable and the lack of stringent filtering can seriously affect subsequent analyses (Korecký et al., 2021). The initial 27,283,139 SNP markers obtained after the reference genome alignment, and implementation of strict filtering criteria helped obtaining high-quality SNP markers and finally only 1.26% of SNPs were retained as high-quality SNP markers. The proportion of retained high-quality SNP markers was much lower than that of other tree species (Mandrou et al., 2014; Tsumura et al., 2020; Yang et al., 2020). And it was found that the highest number of SNP markers but the lowest genetic diversity value was obtained under the first set of criteria (i.e., *MAF* > 0.01, Call rate > 0.9), thus indicating that setting of *MAF* filtering criteria had a greater effect on the number of SNP markers obtained. The filtering criteria for Chinese fir SNP selection in this study were more stringent than those implemented for *Picea abies* (Korecký et al., 2021), *Ulmus pumila* (Lyu et al., 2020), and other Chinese firs (Zheng et al., 2019).

The number of high-quality SNP markers obtained using RAD-seq technology (343,644) was much higher than the number of SNP markers detected by SLAF-seq simplified sequencing technology (108,753/143,871). This may be due to either an increase in sample size (233:221/110) or differences in sequencing technology (Zheng et al., 2019; Huang et al., 2021). RAD-seq sequencing technology not only show high number of markers but also high density (Zhang et al., 2018). This was also observed in some flowers or crops (Jia et al., 2016; Peng et al., 2016; Chankaew et al., 2022; Jiang et al., 2022). The RAD-seq technology often detects more SNPs as compared to SLAF-seq technology (Cai et al., 2015; Su et al., 2017). SNP variant types can be classified into two categories: conversion (Ts) and reversal (Tv), with a theoretical ratio of 0.5. However, a “conversion bias” (Collins and Jukes, 1994) (i.e., conversion/turnover (Ts/Tv) ratio) generally occurs. In this study, before SNP marker screening, the ratio of Ts/Tv was 1.5, whereas it was > 1.5 post screening, with results similar to other findings (Su et al., 2016; Zheng et al., 2019).

### 4.2 The richness of breeding population genetic base

Most coniferous trees have a long growth period, high rate of heterosis, and extensive gene flow, resulting in high level of genetic diversity (Bergmann and Mejnartowicz, 2000). The rich genetic variation within the breeding population forms the basis for genetic improvement (Chaisurisri and El-Kassaby, 1994; El-Kassaby and Ritland, 1996a; El-Kassaby and Ritland, 1996b; Stoehr and El-Kassaby, 1997). The level of population genetic diversity decreases with advanced-generation breeding, as the high intensity of artificial selection generally results in significant short-term genetic gains, while possibly also reducing the genetic variation base and genetic diversity of the breeding population. However, our analysis revealed that the Chinese fir 4^th^ cycle breeding population still harbours high genetic diversity (*P_i_* = 0.003) and high within-population genetic variation, similar to that reported for *Pinus taeda* (Chhatre et al., 2013), *Eucalyptus urophylla* (Yang et al., 2020), *Cryptomeria japonica* (Tsumura et al., 2014), and *Larix kaempferi* (Liu et al., 2017). The introduction of external superior trees (i.e., genetic infusion) leads to increased genetic diversity. Moreover, mating combinations among superior individuals also generate new recombinations, which also results in increased genetic diversity. Additionally, changes in breeding objectives also can increase the genetic variation among populations. The Chinese fir 4^th^ cycle breeding population included not only hybrid offspring between superior trees, but also included external superior trees through genetic infusion. Additionally, the 4^th^ cycle breeding objective added pest resistance attributes to the commonly selected fast-growing, high-quality trees, which may have contributed to the observed high genetic diversity. In addition, some researchers have argued that the Chinese fir germplasm growing in central production areas in suitable environments (e.g., superior seed sources) for long periods is subjected to natural selection, artificial selection, and some anthropogenic activities, leading to the occurrence of pollen and seed exchange and thus gene flow, making it possible for diversity to decrease and the genetic base to narrow (Chen et al., 1980; Li, 2015). The northern Fujian region was considered as one of the central production areas for Chinese fir as early as 20 years ago (Chen et al., 1980; Huang et al., 1986; You and Hong, 1998), and after many years of artificial selection, lower genetic diversity may have occurred, yet high genetic diversity was still detected in seed sources from this region. This may be due to the timely introduction of good external populations to expand the genetic base, and it should also be noted that the northern Fujian seed source also contributed a large number of parents to the Chinese fir breeding population, an observation that supports a previous observation (He, 2019).

The issue of correspondence between the number of parents selected from a particular provenance and genetic diversity (Duan et al., 2017), may suggest that those provenances with a lower number of parents in the breeding population could affect the extent of genetic diversity. Similarly, the AMOVA results showed that over 96% of the genetic variation was present between genotypes, with only very small amount of variation occurring among populations. This was confirmed by the very low *F_st_* values (< 0.05) between subgroups, which may either be related to the unbalanced sample size representation across germplasm origins, or that the parental population was widely used due to its excellent phenotype, and the higher level of human activity may have led to enhancing gene flow, thus reducing genetic differentiation among populations (Fang et al., 2022). This result, which is also consistent with the findings of previous studies, shows that forest trees are predominantly heterozygous and have low genetic differentiation among populations and high levels of overall genetic diversity (Tsumura et al., 2014; Wang et al., 2014; Bínová et al., 2020).

Heterozygosity is an important indicator of the genetic diversity of a population, and the average heterozygosity of the 4^th^ Chinese fir cycle breeding population was high (*H_o_* = 0.215, *H_e_* = 0.233), estimates similar to that reported for the same species (0.163/0.250) (Zheng et al., 2019) and (0.210/0.273) (Huang et al., 2021), *Cryptomeria japonica* (0.269/0.253) (Cai et al., 2020), and also higher than that reported for *Keteleeria davidiana* var. formosana (0.128/0.096) (Shih et al., 2018), *Pinus pungens* (0.113/0.114) and *Pinus rigida* (0.098/0.104) (Bolte et al., 2022), but lower than *Eucalyptus globulus* (0.511/0.423) (Butler et al., 2022), *Pinus strobus* (0.477/0.590) (Whitney et al., 2019), *Cedrus* (0.460/0.530) (Karam et al., 2019). The reasons for the higher heterozygosity estimates in Chinese fir are: 1) highly heterozygous genetic background and broad genetic base, probably due to a long growth cycle, and wind pollination, and 2) the bottleneck effect that may have contributed to high heterozygosity during the Cretaceous to Tertiary Eocene, when the global climate favored the widespread migration of Chinese fir trees between North America and Eurasian continents. During the late Eocene to Oligocene; however, abrupt global climatic changes caused the Chinese fir to disappear from the northern hemisphere at high latitudes. Furthermore, during the Quaternary ice age, the number of Chinese fir trees decreased dramatically, with their distribution becoming smaller and their gradual movement southwards, such that Chinese fir trees were no longer found north of the Qinling and Huai rivers after the Ice Age. Therefore, the Chinese fir may have been affected by the bottleneck effect after the Quaternary ice age, thereby resulting in a sudden increase in heterozygosity followed by a gradual stabilization, with the last ice age also affecting the genetic diversity of species like *Pinus strobus* (Whitney et al., 2019) and *Cryptomeria japonica* (Tsumura et al., 2020) and other tree species. It is also possible that individuals with higher heterozygosity are better suited to survive during evolution, and that the recent selective breeding may also have an effect.

SNP markers detect significantly more genetic variation than SSRs, probably because SNP markers are obtained from the whole genome, have a low genotyping error rate, and have a high density in genomes (Lu et al., 2009)) (e.g., one SNP marker was detected per 46 bp on an average in this study). SNP markers are usually bi-allelic (Vignal et al., 2002), whereas SSR markers are multi-allelic and have a significantly higher number of alleles than SNP markers (Van Inghelandt et al., 2010; Zurn et al., 2020). Studies have shown that double-allelic markers like SNPs can be counted with a maximum genetic diversity of 0.5, whereas multi-allelic markers like SSRs can be observed with genetic diversity values close to 1 (Van Inghelandt et al., 2010). However, some researchers have pointed out that the comparison should not be based only on the number of alleles, but more emphasis must be placed on the number of loci, and that few alleles (but high number of loci with a high gene coverage density) make the estimation of population structure more reliable (Zurn et al., 2020). Genetic diversity parameters obtained from analysis using SNP markers are generally lower than those calculated using traditional molecular markers, like SSR, ISSR, and SRAP (Chen et al., 2017; Duan et al., 2017; Li et al., 2017; García et al., 2018; Lin et al., 2020), which are also similar in other plants (Van Inghelandt et al., 2010; Avican and Bilgen, 2022). Molecular markers can also impact the results of the experiment, as different molecular markers introduce bias in the genetic diversity analysis results for the same or different populations (Bínová et al., 2020; Korecký et al., 2021).

### 4.3 Genetic structure rationalization

The population genetic structure in this study is relatively weak, and aggregation of the same provenances occurs only in some or local branches, which is similar to the findings of Huang et al. (2021) and Xia et al. (2019). The genetic structure of populations is related to a variety of factors, and when the materials are mostly generated from different origins or different geographical sources, the species’ wide range, climate, and complex geography allow for geographical genetic differentiation among the different origins, species sources, or populations, resulting in populations that often have an extremely strong genetic structure fit with geographic sources, like the king of Chinese fir (Li et al., 2016), *Pinus monticola* (Kim et al., 2011), and *Eucalyptus cladocalyx* (Bush and Thumma, 2013; Butler et al., 2022). Chinese fir mainly exists in the southern provinces and regions like Fujian and Guangdong, and the climatic similarity may be the reason for the observed subgrouping. In addition, the large scale long-distance cultivation has increased the genetic exchange among populations, which has gradually increased the complexity of Chinese fir germplasm kinship between different origins, thereby reinforcing the need for molecular techniques for resolving the genetic diversity and population structure (Fang et al., 2022).

Despite the low level of genetic differentiation between breeding generations in Chinese fir, the clustering results for genetic structure suggest it may be related to the genealogical classification and the development of breeding generations, which was similar to the results for significant genetic structure between the 1^st^ and 2^nd^ generation breeding populations of *Pinus taeda* (Chhatre et al., 2013). When the breeding population shows a complex genetic background and is originated from a wide range of sources, its genetic structure will correspond to the kinship between breeding parental sources, as observed in *Eucalyptus urophylla* (Lu et al., 2018). In addition, coniferous trees usually have low levels of genetic differentiation due to heterosis and gradual gene penetration (Petit and Hampe, 2006), e.g. no significant population structure was detected within *Pinus pungens* and *P. rigida* based on the whole genome-wide data (Bolte et al., 2022).

The observed clustering results of the Chinese fir 4^th^ cycle breeding population may also be related to the three previous recurrent selection cycles. The 1^st^ cycle breeding population dates back to the 1860s. However, over the years, the breeding objectives have mainly targeted fast growth and productivity, with the 4^th^ cycle breeding population being selected for fast-growing, high quality, and stress resistance. This may result in some of the Chinese fir germplasm parental trees being repeatedly selected as mating parents due to their excellent performance. Repeated artificial selection may gradually intensify the performance of the target traits, thereby increasing the frequency of related advantageous loci, which may further produce a linkage disequilibrium effect and make the genetic structure of the artificially improved breeding populations likewise differ significantly (Du et al., 2021), so exploring population genetic structure should be considered from multiple aspects and dimensions, not just individual condition such as geographical factors or genealogical structure.

## 5 Conclusion

In this paper, we made a preliminary attempt to construct a reference genome for Chinese fir using RAD-seq. We genotyped 233 parents and the development of a large number of (343,644) high-quality SNP markers. Furthermore, we detected that the genetic diversity of the 4^th^ cycle breeding population was abundant. The genetic differentiation among populations was not obvious, leading to no apparent population structure. Most of the observed variation mainly originated among individuals, which may be related to the frequent exchange between Chinese fir origins and its long history of cultivation and domestication. Therefore, population structure is not significantly correlated with germplasm origin, but may be related to the genealogy and breeding generation.

## Data availability statement

The datasets presented in this study can be found in online repositories. The name of the repository and accession numbers can be found below: NCBI; PRJNA910811 and PRJNA909424.

## Author contributions

YJ, LB and XFZ contributed to conception and design of the study. YJ, LB, XFZ, BZ, RZ, SS, DY and XYZ organized the database. YJ, LB, XFZ and BZ performed the statistical analysis. YJ, LB and XFZ wrote the first draft of the manuscript. YE-K and JS wrote sections of the manuscript. All authors contributed to the article and approved the submitted version.
