# Supplementary material for: Genetic diversity and structure of the 4th cycle breeding population of Chinese fir (Cunninghamia lanceolata (lamb.) hook)
Source: Front Plant Sci. 2023 Jan 27;14:1106615. doi: 10.3389/fpls.2023.1106615 (PMC9911867; doi:10.3389/fpls.2023.1106615)
Supplement: Supplementary file 1 [file DataSheet_1.docx]

Supplementary Material

**Supplementary Figure 1.** The Chinease fir germplasm phylogenetic tree. FJ, HN, SC, JX, GD, XQG, SYE, and MO denote Fujian, Hunan, Sichuan, Jiangxi, Guangdong, Hunan-Guizhou-Guangxi border, Shaanxi-Henan-Hubei border, and mixed provenance, respectively.


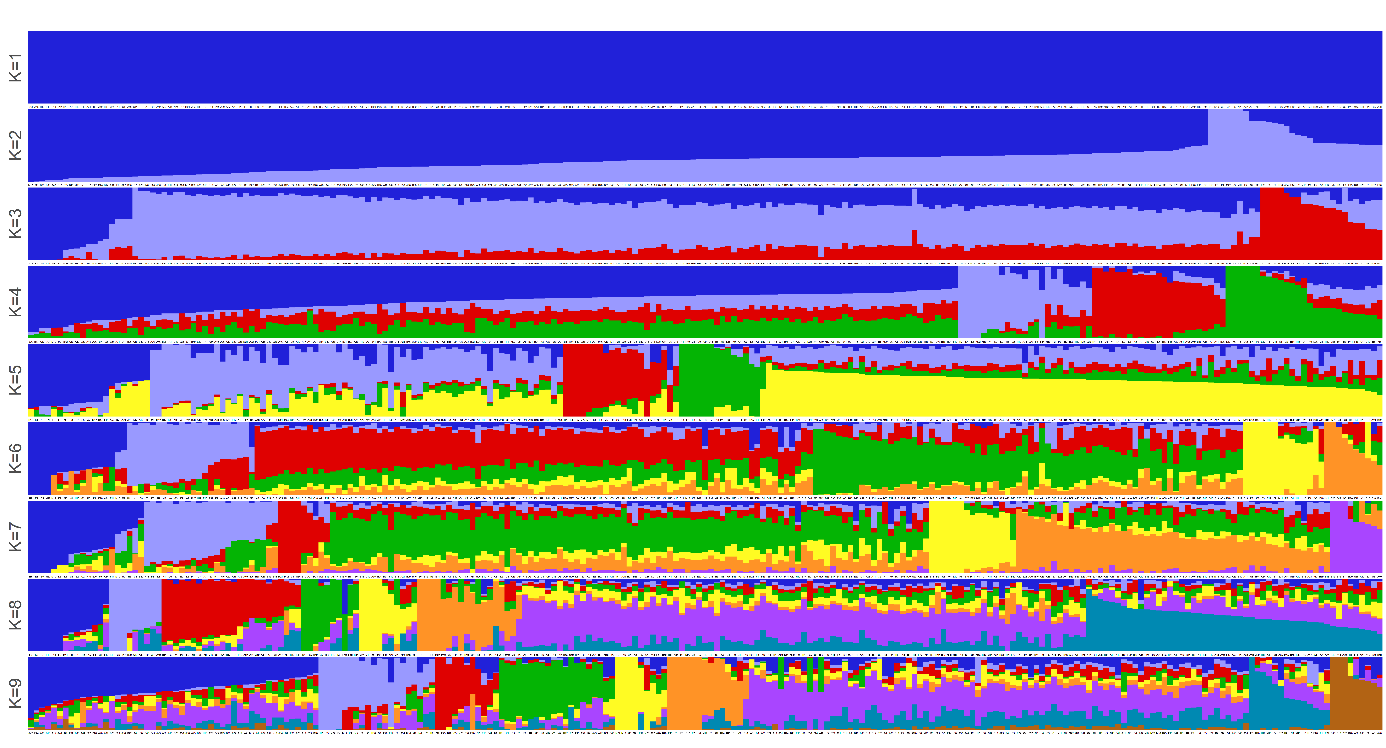


**Supplementary Figure 2.** Population structure of 233 Chinese fir germplasm.

**Supplementary Figure 3.** Population structure of 233 Chinese fir germplasm in four generations. G1~G4 represent the 1^st^, 2^nd^, 3^rd^ and 4^th^ generation breeding parents, respectively.


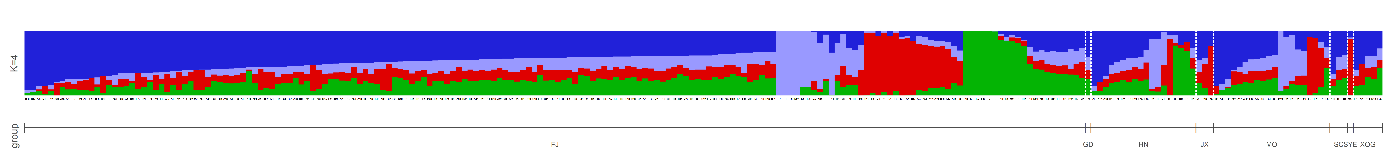


**Supplementary Figure 4.** Population structure of 233 Chinese fir germplasm from different provenances. FJ, HN, SC, JX, GD, XQG, SYE, and MO denote Fujian, Hunan, Sichuan, Jiangxi, Guangdong, Hunan-Guizhou-Guangxi border, Shaanxi-Henan-Hubei border, and mixed provenance, respectively.

| a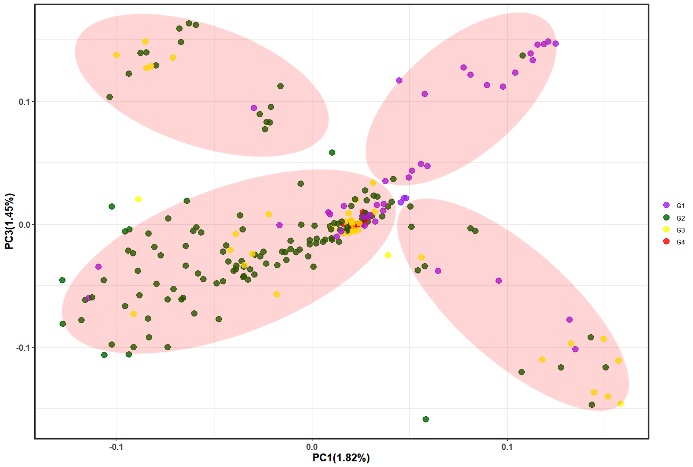 | b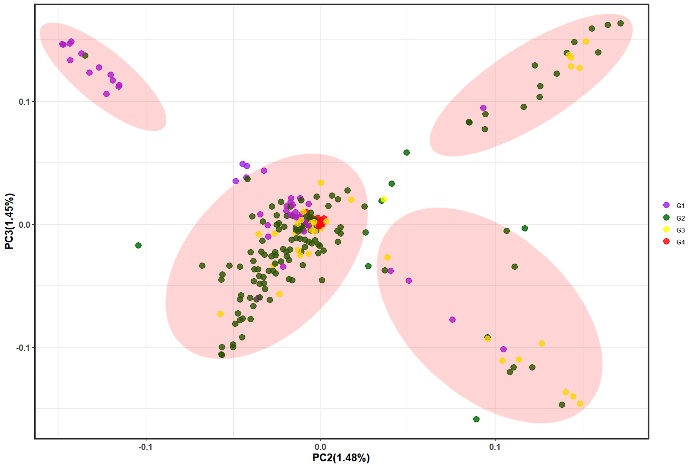 |
| --- | --- |
| c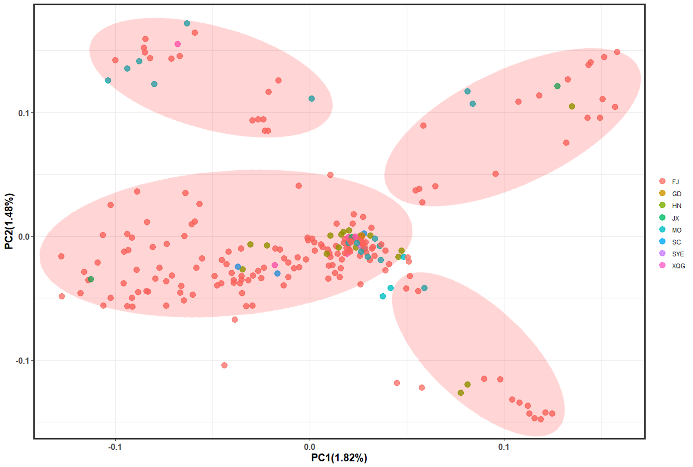 | d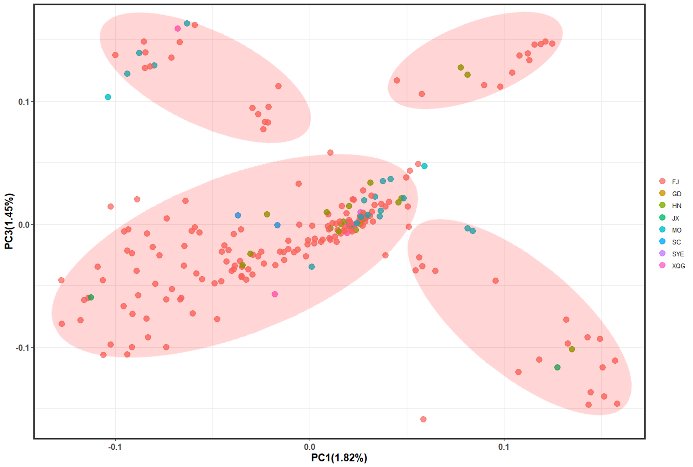 |
| e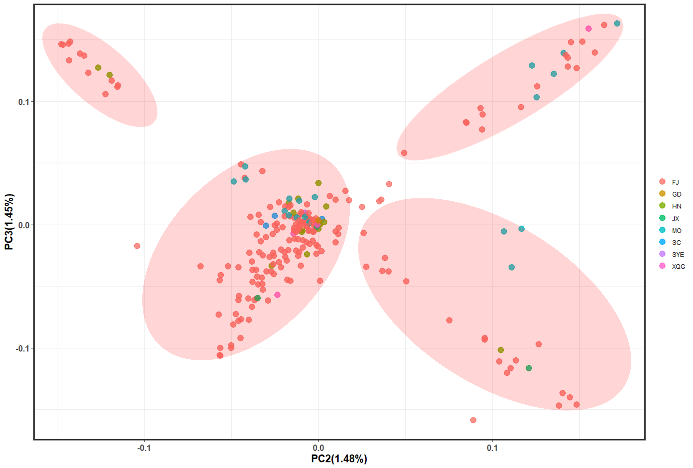 |  |

**Supplementary Figure 5.** Principal Component Analysis **a** and **b** Different generations **c,d** and **e** Different provenances. PC1, PC2 and PC3 represent the first, second and third principal components, respectively. G1~G4 represent the 1^st^, 2^nd^, 3^rd^ and 4^th^ generation breeding parents, respectively. FJ, HN, SC, JX, GD, XQG, SYE, and MO denote Fujian, Hunan, Sichuan, Jiangxi, Guangdong, Hunan-Guizhou-Guangxi border, Shaanxi-Henan-Hubei border, and mixed provenance, respectively.

**Supplementary Tables 1.** 233 individuals of Chiness fir from generation 1 to generation 4 used in this trial.

| **Classification** | | **Germplasm number and source** | **quantity** |
| --- | --- | --- | --- |
| G1 | FJ:H2, H3, E1, H12, H13, E11, E12, F5, F6, E13, F7, F8, E14, F9, F10, K6, H15, K7, M11, M12, M25, E22, E25, E28, H23, G6, G7, G8, M28;  HN:K5, H14, E16, M13, G1, G2, G4;  SC:E21;  MO:H5, K2, K11, G5, M26, M27; | | 43 |
| G2 | FJ: B1, B2, B3, B4, B5, B6, B7, B8, A1, B9, B10, B11, B12, A2, A3, A4, A5, A6, A7, A8, A9, A10, B14, A11, B15, A12, A13, B16, B17, A14, A16, A17, A18, A19, A20, B18, B19, B20, A22, B21, A23, A24, A25, B22, B23, A26, A27, B24, B25, B26, B27, A28, A29, A30, A31, A32, A33, A34, A38, A39, A40, A41, B28, A42, A43, B29, B30, B31, B33, B35, B36, B37, B38, B39, B40, B41, A44, A45, A46, A47, A48, A49, A51, A52, B42, B43, B44, B45, B46, B47, B48, B49, H4, E3, M7, F1, F3, E8, E10, E15, F11, N2, E17, H19, M14, N3, M15, M19, M21, M22, M24, F13, E19, H20, E20, E23, E24, E26, F14, H22, K9, F16, K10;  HN: E27;  SC: M2;  JX: E2, M3;  XQG: K1;  MO: H1, E6, E7, M1, M8, M9, M10, H11, E9, F12, H17, F15, H21; | | 141 |
| G3 | FJ: A15, A35, B32, A50, C12, C13, H6, H7, E4, E5, H8, H9, M4, M5, M6, N1, F4, H10, K3, K4, H18, E18, M16, M17, M18, M20, K8, G3;  HN: B13, A21, B34, A53, F2, H16, M23;  XQG: A36, A37;  MO: G9; | | 38 |
| G4 | FJ: C2, C6;  HN: C1, C10, C11;  SC: C8;  JX: C4;  GD: C9;  XQG: C3, C7;  SYE: C5; | | 11 |
| Total |  | | 233 |

Note: G1~G4 represent the 1^st^, 2^nd^, 3^rd^ and 4^th^ generation breeding parents, respectively. FJ, HN, SC, JX, GD, XQG, SYE, and MO denote Fujian, Hunan, Sichuan, Jiangxi, Guangdong, Hunan-Guizhou-Guangxi border, Shaanxi-Henan-Hubei border, and mixed provenance, respectively.

**Supplementary Tables 2.** Pre-filter statistics for sequencing data.

| **ID** | **sample** | **RawData(bp)** | **BF_Q20(%)** | **BF_Q30(%)** | **BF_N(%)** | **BF_GC(%)** |
| --- | --- | --- | --- | --- | --- | --- |
| C3 | E149 | 15938412952 | 15496376942 (97.23%) | 14696531669 (92.21%) | 1185579 (0.01%) | 6032597778 (37.85%) |
| K4 | E35 | 14185661636 | 13901783562 (98.00%) | 13335113031 (94.00%) | 63488 (0.00%) | 5390219690 (38.00%) |
| B15 | E84 | 10677953820 | 10392810350 (97.33%) | 9855165339 (92.29%) | 13858 (0.00%) | 4028587057 (37.73%) |
| H9 | C2 | 14720907272 | 14411926559 (97.90%) | 13809276990 (93.81%) | 62536 (0.00%) | 5575374314 (37.87%) |
| E23 | C18 | 10321271440 | 10039480247 (97.27%) | 9529277284 (92.33%) | 76343 (0.00%) | 3897659524 (37.76%) |
| H17 | E41 | 13151035556 | 12730626139 (96.80%) | 12019996845 (91.40%) | 94614 (0.00%) | 4966948883 (37.77%) |
| F2 | E24 | 10748827768 | 10478321885 (97.48%) | 9964478364 (92.70%) | 32089 (0.00%) | 4064083714 (37.81%) |
| E24 | E59 | 11613922444 | 11277892011 (97.11%) | 10659774951 (91.78%) | 9943 (0.00%) | 4372322092 (37.65%) |
| F4 | E25 | 13389017600 | 12923931206 (96.53%) | 12159640202 (90.82%) | 134402 (0.00%) | 5082727864 (37.96%) |
| E25 | E60 | 12285297604 | 11910763511 (96.95%) | 11264417336 (91.69%) | 35175 (0.00%) | 4630681541 (37.69%) |
| E28 | E61 | 13019493644 | 12514504487 (96.12%) | 11736946711 (90.15%) | 671494 (0.01%) | 4903919822 (37.67%) |
| C6 | E152 | 16468810220 | 15954062448 (96.87%) | 15086266008 (91.61%) | 119655 (0.00%) | 6207920000 (37.70%) |
| H15 | E38 | 12925388760 | 12572332457 (97.27%) | 11917705688 (92.20%) | 10917 (0.00%) | 4870101644 (37.68%) |
| F7 | B6 | 11560222768 | 11245720070 (97.28%) | 10670838248 (92.31%) | 9932 (0.00%) | 4365549978 (37.76%) |
| C5 | E151 | 15255295888 | 14768199715 (96.81%) | 13935588259 (91.35%) | 43636 (0.00%) | 5730590238 (37.56%) |
| K8 | E56 | 10684198240 | 10321935908 (96.61%) | 9733083809 (91.10%) | 117411 (0.00%) | 4037575034 (37.79%) |
| E27 | B13 | 9962222400 | 9649086368 (96.86%) | 9100328490 (91.35%) | 118003 (0.00%) | 3759602536 (37.74%) |
| E17 | E42 | 9941876132 | 9581580769 (96.38%) | 9012514853 (90.65%) | 509986 (0.01%) | 3739533061 (37.61%) |
| F10 | E33 | 11374838684 | 10911502453 (95.93%) | 10208897686 (89.75%) | 597230 (0.01%) | 4300506965 (37.81%) |
| E19 | E55 | 10778182528 | 10516634882 (97.57%) | 10002637655 (92.80%) | 32584 (0.00%) | 4052371117 (37.60%) |
| E20 | E57 | 12719415172 | 12439093302 (97.80%) | 11861536960 (93.26%) | 148579 (0.00%) | 4783758353 (37.61%) |
| C8 | E154 | 13705519028 | 13176201658 (96.14%) | 12361060215 (90.19%) | 691515 (0.01%) | 5147357316 (37.56%) |
| K7 | E39 | 14057701688 | 13647935643 (97.09%) | 12917156320 (91.89%) | 757729 (0.01%) | 5307183401 (37.75%) |
| K5 | B10 | 13433769228 | 13075277489 (97.33%) | 12438636488 (92.59%) | 121592 (0.00%) | 5075852017 (37.78%) |
| C2 | E148 | 12552891076 | 12175227310 (96.99%) | 11521813497 (91.79%) | 202270 (0.00%) | 4712897631 (37.54%) |
| E22 | C17 | 11910529620 | 11542598417 (96.91%) | 10903735328 (91.55%) | 191577 (0.00%) | 4472353221 (37.55%) |
| K2 | B8 | 13317553812 | 12991717642 (97.55%) | 12386196553 (93.01%) | 375321 (0.00%) | 5013116294 (37.64%) |
| A1 | E69 | 11455933216 | 11145476470 (97.29%) | 10560982689 (92.19%) | 48818 (0.00%) | 4283102425 (37.39%) |
| C7 | E153 | 12037475744 | 11655879120 (96.83%) | 10991363348 (91.31%) | 147191 (0.00%) | 4520040629 (37.55%) |
| C1 | E147 | 13590261956 | 13137013764 (96.66%) | 12373664783 (91.05%) | 139094 (0.00%) | 5099969670 (37.53%) |
| K9 | E63 | 12150146864 | 11910583829 (98.03%) | 11402342031 (93.85%) | 78493 (0.00%) | 4542898015 (37.39%) |
| B48 | E145 | 15163105940 | 14842828051 (97.89%) | 14195364254 (93.62%) | 107932 (0.00%) | 5677969306 (37.45%) |
| F3 | C6 | 14364453820 | 14042880504 (97.76%) | 13397685763 (93.27%) | 169027 (0.00%) | 5410619128 (37.67%) |
| F5 | E30 | 14609390428 | 14169781557 (96.99%) | 13428675035 (91.92%) | 231150 (0.00%) | 5524042012 (37.81%) |
| A2 | E74 | 16608870356 | 16115180137 (97.03%) | 15222597627 (91.65%) | 316950 (0.00%) | 6212205331 (37.40%) |
| G4 | F8 | 13425724336 | 13003198468 (96.85%) | 12281972424 (91.48%) | 97549 (0.00%) | 5030283973 (37.47%) |
| C4 | E150 | 11701085904 | 11311384751 (96.67%) | 10676868627 (91.25%) | 100067 (0.00%) | 4382252756 (37.45%) |
| F11 | A2 | 12438206324 | 11986810679 (96.37%) | 11284181810 (90.72%) | 632200 (0.01%) | 4675344039 (37.59%) |
| B16 | E87 | 19281334948 | 18568397826 (96.30%) | 17369466924 (90.08%) | 280994 (0.00%) | 7213811430 (37.41%) |
| M5 | E17 | 14388186412 | 13861979165 (96.34%) | 13031590849 (90.57%) | 138262 (0.00%) | 5390867061 (37.47%) |
| M4 | E16 | 11920231028 | 11696541465 (98.12%) | 11219011844 (94.12%) | 53892 (0.00%) | 4455630715 (37.38%) |
| E21 | E58 | 13470069792 | 13014753155 (96.62%) | 12249602648 (90.94%) | 138522 (0.00%) | 5055866176 (37.53%) |
| H13 | B2 | 15102292268 | 14762599565 (97.75%) | 14063363113 (93.12%) | 114091 (0.00%) | 5671795492 (37.56%) |
| A5 | E77 | 12070346768 | 11764844550 (97.47%) | 11182213063 (92.64%) | 11277 (0.00%) | 4508761554 (37.35%) |
| K6 | E36 | 10201831760 | 9779598964 (95.86%) | 9140987875 (89.60%) | 519434 (0.01%) | 3850951479 (37.75%) |
| M2 | E14 | 14378994544 | 14033334165 (97.60%) | 13368087905 (92.97%) | 231692 (0.00%) | 5369915139 (37.35%) |
| F6 | B4 | 12053758832 | 11723086096 (97.26%) | 11145901991 (92.47%) | 90296 (0.00%) | 4553741044 (37.78%) |
| B49 | E146 | 17313953412 | 16972677012 (98.03%) | 16244432938 (93.82%) | 189273 (0.00%) | 6480209691 (37.43%) |
| B19 | E96 | 13724583124 | 13321647194 (97.06%) | 12614543128 (91.91%) | 49102 (0.00%) | 5126335520 (37.35%) |
| B23 | E101 | 16093026076 | 15629715653 (97.12%) | 14773354043 (91.80%) | 1300029 (0.01%) | 6010729896 (37.35%) |
| A45 | E132 | 12534903876 | 12169827723 (97.09%) | 11489192872 (91.66%) | 37076 (0.00%) | 4677964542 (37.32%) |
| H14 | E37 | 15043443756 | 14673233385 (97.54%) | 13988140541 (92.98%) | 415444 (0.00%) | 5647640347 (37.54%) |
| G9 | F13 | 14856296576 | 14460130010 (97.33%) | 13734446187 (92.45%) | 75916 (0.00%) | 5561715733 (37.44%) |
| H10 | C7 | 12186390488 | 11943085995 (98.00%) | 11439965389 (93.87%) | 79170 (0.00%) | 4572949858 (37.53%) |
| F15 | C15 | 12882212180 | 12596255912 (97.78%) | 12012108455 (93.25%) | 150365 (0.00%) | 4819192522 (37.41%) |
| B13 | E79 | 13300957408 | 12962322445 (97.45%) | 12319825625 (92.62%) | 12682 (0.00%) | 4967372472 (37.35%) |
| B42 | E139 | 11990407972 | 11735725170 (97.88%) | 11218617449 (93.56%) | 86609 (0.00%) | 4471457816 (37.29%) |
| E26 | C19 | 16985224484 | 16449304471 (96.84%) | 15529279358 (91.43%) | 123931 (0.00%) | 6361296098 (37.45%) |
| H11 | E26 | 15742170848 | 15345147022 (97.48%) | 14601502357 (92.75%) | 79243 (0.00%) | 5890345543 (37.42%) |
| F1 | E23 | 14956181600 | 14530172073 (97.15%) | 13768732621 (92.06%) | 55320 (0.00%) | 5587662360 (37.36%) |
| K10 | F3 | 11087374904 | 10852882574 (97.89%) | 10381563655 (93.63%) | 307195 (0.00%) | 4138688799 (37.33%) |
| B24 | E103 | 14660136524 | 14266333941 (97.31%) | 13536779243 (92.34%) | 13606 (0.00%) | 5466933937 (37.29%) |
| E15 | E34 | 16371468516 | 15893114436 (97.08%) | 15041984254 (91.88%) | 120747 (0.00%) | 6117202291 (37.37%) |
| B27 | E106 | 16299994508 | 15734352526 (96.53%) | 14753563643 (90.51%) | 179756 (0.00%) | 6076955681 (37.28%) |
| H8 | E11 | 11666454120 | 11216283482 (96.14%) | 10529334918 (90.25%) | 582159 (0.00%) | 4388361117 (37.62%) |
| A47 | E134 | 14781684736 | 14132670887 (95.61%) | 13129621028 (88.82%) | 148881 (0.00%) | 5510289800 (37.28%) |
| B14 | E82 | 10973779896 | 10686354852 (97.38%) | 10142706152 (92.43%) | 133441 (0.00%) | 4091345336 (37.28%) |
| H4 | E4 | 12425719820 | 12072861377 (97.16%) | 11442632595 (92.09%) | 10866 (0.00%) | 4675045828 (37.62%) |
| A49 | E135 | 13955721856 | 13579761501 (97.31%) | 12880947283 (92.30%) | 13034 (0.00%) | 5201454578 (37.27%) |
| C9 | E155 | 11395842536 | 11051996784 (96.98%) | 10462945933 (91.81%) | 41646 (0.00%) | 4256300179 (37.35%) |
| B38 | C35 | 13275334992 | 12763736774 (96.15%) | 11926514655 (89.84%) | 217099 (0.00%) | 4955126196 (37.33%) |
| H23 | F1 | 18378185372 | 17949147709 (97.67%) | 17072780855 (92.90%) | 139602 (0.00%) | 6884780128 (37.46%) |
| B18 | C26 | 12161617500 | 11777640647 (96.84%) | 11104353911 (91.31%) | 656803 (0.01%) | 4533075684 (37.27%) |
| F13 | E54 | 11460976348 | 11188322249 (97.62%) | 10655509215 (92.97%) | 33872 (0.00%) | 4278086884 (37.33%) |
| M22 | C13 | 10956686800 | 10693621058 (97.60%) | 10166318587 (92.79%) | 280045 (0.00%) | 4083237427 (37.27%) |
| F8 | B7 | 12562873096 | 12182220180 (96.97%) | 11524322332 (91.73%) | 37707 (0.00%) | 4697280790 (37.39%) |
| B35 | E127 | 11806024864 | 11444180519 (96.94%) | 10816299649 (91.62%) | 86715 (0.00%) | 4397495111 (37.25%) |
| G7 | F11 | 17488877180 | 17143466823 (98.02%) | 16445959012 (94.04%) | 72457 (0.00%) | 6520162687 (37.28%) |
| B41 | E130 | 14212832236 | 13767887090 (96.87%) | 13016995763 (91.59%) | 50886 (0.00%) | 5295336650 (37.26%) |
| B9 | E70 | 16427991540 | 15941049774 (97.04%) | 15041649323 (91.56%) | 299455 (0.00%) | 6119429841 (37.25%) |
| H21 | A7 | 13688029396 | 13410253261 (97.97%) | 12841049043 (93.81%) | 86845 (0.00%) | 5122213610 (37.42%) |
| B25 | E104 | 11187483600 | 10899631624 (97.43%) | 10350876305 (92.52%) | 135245 (0.00%) | 4165980951 (37.24%) |
| G6 | F10 | 11906606892 | 11479228396 (96.41%) | 10799845138 (90.70%) | 593103 (0.00%) | 4442277043 (37.31%) |
| M26 | F14 | 13375182056 | 13125277616 (98.13%) | 12585911227 (94.10%) | 60369 (0.00%) | 4977418888 (37.21%) |
| F12 | A3 | 15496334880 | 15167336672 (97.88%) | 14525724737 (93.74%) | 64861 (0.00%) | 5781527422 (37.31%) |
| B2 | C21 | 12177078608 | 11899707840 (97.72%) | 11351518681 (93.22%) | 11338 (0.00%) | 4534179867 (37.24%) |
| M24 | E52 | 12966846920 | 12662707868 (97.65%) | 12070926395 (93.09%) | 207958 (0.00%) | 4826599322 (37.22%) |
| A42 | E119 | 15592810804 | 15142433027 (97.11%) | 14284884076 (91.61%) | 341163 (0.00%) | 5802202342 (37.21%) |
| M3 | E15 | 11635182380 | 11224913192 (96.47%) | 10568175824 (90.83%) | 836595 (0.01%) | 4337049533 (37.28%) |
| H19 | B11 | 11241521120 | 10831269615 (96.35%) | 10186055021 (90.61%) | 575931 (0.01%) | 4210108322 (37.45%) |
| H16 | E40 | 13136319340 | 12750394741 (97.06%) | 12074669886 (91.92%) | 37484 (0.00%) | 4898497672 (37.29%) |
| A44 | E131 | 19078320780 | 18438333494 (96.65%) | 17366322082 (91.03%) | 68054 (0.00%) | 7101436090 (37.22%) |
| A37 | E113 | 12263188532 | 11843327455 (96.58%) | 11145957249 (90.89%) | 1427410 (0.01%) | 4564016994 (37.22%) |
| F14 | B12 | 12332109000 | 11913392737 (96.60%) | 11206889369 (90.88%) | 124910 (0.00%) | 4596469481 (37.27%) |
| A16 | E90 | 15341180680 | 14967415349 (97.56%) | 14248116011 (92.87%) | 14521 (0.00%) | 5706680411 (37.20%) |
| B10 | E71 | 14137964604 | 13791223435 (97.55%) | 13092650440 (92.61%) | 84562 (0.00%) | 5258776154 (37.20%) |
| H5 | E5 | 12939998980 | 12522039046 (96.77%) | 11816567563 (91.32%) | 37722 (0.00%) | 4850130095 (37.48%) |
| E1 | E3 | 19406625140 | 18801541161 (96.88%) | 17732244263 (91.37%) | 234385 (0.00%) | 7221185012 (37.21%) |
| B17 | E88 | 14171964500 | 13632190550 (96.19%) | 12726536771 (89.80%) | 171032 (0.00%) | 5272958252 (37.21%) |
| H20 | C16 | 17719303724 | 17356996601 (97.96%) | 16638963660 (93.90%) | 74843 (0.00%) | 6613652324 (37.32%) |
| H1 | E1 | 17147854468 | 16740932390 (97.63%) | 15918181492 (92.83%) | 129412 (0.00%) | 6405258753 (37.35%) |
| C11 | E157 | 15901097688 | 15594833257 (98.07%) | 14923009842 (93.85%) | 174736 (0.00%) | 5920786498 (37.24%) |
| H12 | E27 | 16837561544 | 16462196308 (97.77%) | 15673563569 (93.09%) | 127993 (0.00%) | 6273710487 (37.26%) |
| B7 | E67 | 13361577192 | 12942808718 (96.87%) | 12203129559 (91.33%) | 712664 (0.01%) | 4968290157 (37.18%) |
| B1 | C20 | 11899559764 | 11555305412 (97.11%) | 10922548833 (91.79%) | 191822 (0.00%) | 4421878217 (37.16%) |
| B8 | E68 | 13382839464 | 12977512883 (96.97%) | 12273014958 (91.71%) | 48060 (0.00%) | 4974779858 (37.17%) |
| E8 | C8 | 11119707188 | 10852282516 (97.60%) | 10322245478 (92.83%) | 33642 (0.00%) | 4136466898 (37.20%) |
| M19 | E51 | 10021615492 | 9677033537 (96.56%) | 9107507797 (90.88%) | 111750 (0.00%) | 3729242171 (37.21%) |
| A7 | C24 | 17103868756 | 16670624877 (97.47%) | 15831606290 (92.56%) | 21354 (0.00%) | 6352701393 (37.14%) |
| B40 | C36 | 11330948164 | 10969592371 (96.81%) | 10349204964 (91.34%) | 615663 (0.01%) | 4210285265 (37.16%) |
| B12 | E73 | 16511869124 | 15969141827 (96.71%) | 15013693794 (90.93%) | 439641 (0.00%) | 6136230067 (37.16%) |
| A53 | E143 | 14900661888 | 14388648580 (96.56%) | 13507793862 (90.65%) | 152164 (0.00%) | 5534162472 (37.14%) |
| M13 | E46 | 12412085756 | 12122335611 (97.67%) | 11557984028 (93.12%) | 202615 (0.00%) | 4611964109 (37.16%) |
| M16 | A6 | 15706472972 | 15327466516 (97.59%) | 14578769877 (92.82%) | 146685 (0.00%) | 5840697291 (37.19%) |
| B3 | E64 | 10665143780 | 10405992401 (97.57%) | 9899855443 (92.82%) | 129188 (0.00%) | 3962107978 (37.15%) |
| B26 | E105 | 18345044832 | 17882268890 (97.48%) | 16986597039 (92.60%) | 1599768 (0.01%) | 6812317844 (37.13%) |
| E12 | E29 | 11161854760 | 10869306550 (97.38%) | 10320923817 (92.47%) | 82742 (0.00%) | 4148888017 (37.17%) |
| F16 | F2 | 13804396360 | 13409570010 (97.14%) | 12712831290 (92.09%) | 221287 (0.00%) | 5145341564 (37.27%) |
| K1 | E18 | 16490529472 | 16117645468 (97.74%) | 15351550495 (93.09%) | 125077 (0.00%) | 6161013236 (37.36%) |
| A9 | E80 | 17649841596 | 17167231188 (97.27%) | 16224724506 (91.93%) | 385185 (0.00%) | 6551945291 (37.12%) |
| A15 | E89 | 13046707752 | 12660866768 (97.04%) | 11981500251 (91.84%) | 1523281 (0.01%) | 4844948755 (37.14%) |
| A38 | E114 | 15374859668 | 14987505020 (97.48%) | 14251556844 (92.69%) | 14811 (0.00%) | 5707338244 (37.12%) |
| E13 | B5 | 11843453716 | 11530262836 (97.36%) | 10934031677 (92.32%) | 10326 (0.00%) | 4405983352 (37.20%) |
| B30 | E122 | 13331259416 | 12922177913 (96.93%) | 12214314840 (91.62%) | 47648 (0.00%) | 4948181017 (37.12%) |
| B34 | E126 | 11220015028 | 10879182605 (96.96%) | 10278205512 (91.61%) | 179504 (0.00%) | 4164923123 (37.12%) |
| B43 | C38 | 15984401200 | 15641543262 (97.86%) | 14922792015 (93.36%) | 174878 (0.00%) | 5926975198 (37.08%) |
| M23 | C14 | 13938814180 | 13599701589 (97.57%) | 12954686253 (92.94%) | 225093 (0.00%) | 5177308733 (37.14%) |
| A46 | E133 | 13062983540 | 12706750906 (97.27%) | 12010519709 (91.94%) | 78871 (0.00%) | 4843148417 (37.08%) |
| E4 | E9 | 16210669956 | 15707436792 (96.90%) | 14832749316 (91.50%) | 119463 (0.00%) | 6024552894 (37.16%) |
| A48 | C37 | 13553442216 | 13125295152 (96.84%) | 12373499455 (91.29%) | 217902 (0.00%) | 5025686862 (37.08%) |
| C12 | E158 | 12527311584 | 12110347778 (96.67%) | 11389316689 (90.92%) | 127798 (0.00%) | 4653695787 (37.15%) |
| B44 | E140 | 12821097456 | 12318224207 (96.08%) | 11512595648 (89.79%) | 130363 (0.00%) | 4755748185 (37.09%) |
| N3 | E48 | 15382078492 | 15019733302 (97.64%) | 14316843487 (93.07%) | 78334 (0.00%) | 5704023243 (37.08%) |
| A4 | E76 | 10855627060 | 10515741190 (96.87%) | 9932565638 (91.50%) | 1274183 (0.01%) | 4028019744 (37.11%) |
| B36 | C34 | 10730690772 | 10366440249 (96.61%) | 9778200996 (91.12%) | 93177 (0.00%) | 3990083105 (37.18%) |
| E5 | E10 | 10170887936 | 9833782886 (96.69%) | 9240276159 (90.85%) | 123703 (0.00%) | 3775495991 (37.12%) |
| B6 | E66 | 14968687084 | 14454768296 (96.57%) | 13558605157 (90.58%) | 180183 (0.00%) | 5553682648 (37.10%) |
| A6 | C23 | 10659801932 | 10369132807 (97.27%) | 9830765231 (92.22%) | 128866 (0.00%) | 3951547506 (37.07%) |
| F9 | E32 | 12590525788 | 12203678670 (96.93%) | 11533650761 (91.61%) | 91569 (0.00%) | 4677674269 (37.15%) |
| A43 | E120 | 11828772832 | 11440485506 (96.72%) | 10769287244 (91.04%) | 638421 (0.01%) | 4386647080 (37.08%) |
| B21 | C27 | 12039234460 | 11733544992 (97.46%) | 11128128915 (92.43%) | 71910 (0.00%) | 4460345558 (37.05%) |
| N1 | C5 | 14504170856 | 14125375523 (97.39%) | 13399756130 (92.39%) | 806216 (0.01%) | 5381224210 (37.10%) |
| M21 | C12 | 12194559188 | 11910389579 (97.67%) | 11355653559 (93.12%) | 345886 (0.00%) | 4518595540 (37.05%) |
| M12 | C10 | 12553525300 | 12253997807 (97.61%) | 11676811523 (93.02%) | 202604 (0.00%) | 4654868659 (37.08%) |
| H22 | E62 | 13345762764 | 13017829947 (97.54%) | 12397042022 (92.89%) | 68280 (0.00%) | 4966991116 (37.22%) |
| A52 | E138 | 14232435072 | 13896128859 (97.64%) | 13210187098 (92.82%) | 85716 (0.00%) | 5275146523 (37.06%) |
| M7 | E19 | 12721487788 | 12384080334 (97.35%) | 11736523561 (92.26%) | 703203 (0.01%) | 4714411003 (37.06%) |
| A31 | C32 | 15690014392 | 15262758673 (97.28%) | 14428385708 (91.96%) | 342018 (0.00%) | 5811578180 (37.04%) |
| C13 | E159 | 12541590968 | 12172869920 (97.06%) | 11509544612 (91.77%) | 200318 (0.00%) | 4651444872 (37.09%) |
| N2 | C9 | 13933561976 | 13488246294 (96.80%) | 12732085479 (91.38%) | 153063 (0.00%) | 5168518759 (37.09%) |
| B33 | E125 | 13059636928 | 12598986686 (96.47%) | 11825271732 (90.55%) | 135386 (0.00%) | 4839964931 (37.06%) |
| C10 | E156 | 11474874088 | 11241224127 (97.96%) | 10761197063 (93.78%) | 82816 (0.00%) | 4260947808 (37.13%) |
| K11 | F5 | 10693001748 | 10448898861 (97.72%) | 9957615235 (93.12%) | 276700 (0.00%) | 3964167820 (37.07%) |
| M27 | F15 | 11669937388 | 11376640664 (97.49%) | 10817468245 (92.70%) | 104561 (0.00%) | 4325951725 (37.07%) |
| A51 | E137 | 10459188296 | 10204824529 (97.57%) | 9697363293 (92.72%) | 30702 (0.00%) | 3873475904 (37.03%) |
| A40 | E116 | 16427776044 | 15989907323 (97.33%) | 15157771692 (92.27%) | 20599 (0.00%) | 6085467937 (37.04%) |
| A12 | E85 | 10955348272 | 10660616273 (97.31%) | 10105407153 (92.24%) | 47370 (0.00%) | 4057153517 (37.03%) |
| E7 | E13 | 12204628516 | 11851341426 (97.11%) | 11238807727 (92.09%) | 43608 (0.00%) | 4540029363 (37.20%) |
| E16 | A4 | 11322679308 | 10988749013 (97.05%) | 10380202065 (91.68%) | 135537 (0.00%) | 4208657717 (37.17%) |
| A32 | E109 | 12334317688 | 11930854531 (96.73%) | 11237855588 (91.11%) | 688649 (0.01%) | 4568709474 (37.04%) |
| B5 | E65 | 15468552832 | 14984499789 (96.87%) | 14105690647 (91.19%) | 1182905 (0.01%) | 5735110946 (37.08%) |
| E11 | B3 | 13264129200 | 12892046559 (97.19%) | 12216728002 (92.10%) | 212938 (0.00%) | 4923894451 (37.12%) |
| A50 | E136 | 13462427276 | 13059522570 (97.01%) | 12342073819 (91.68%) | 162919 (0.00%) | 4984345640 (37.02%) |
| A36 | E112 | 11599543780 | 11236535464 (96.87%) | 10611491170 (91.48%) | 1373157 (0.01%) | 4296120155 (37.04%) |
| B47 | E144 | 10668808088 | 10307653571 (96.61%) | 9722769463 (91.13%) | 91167 (0.00%) | 3951045358 (37.03%) |
| G8 | F12 | 13671347436 | 13378070947 (97.85%) | 12788227676 (93.54%) | 87671 (0.00%) | 5074273713 (37.12%) |
| A11 | E83 | 14636845728 | 14229658970 (97.22%) | 13491423144 (92.17%) | 51867 (0.00%) | 5419845510 (37.03%) |
| M20 | C11 | 14958354956 | 14618412514 (97.73%) | 13951821841 (93.27%) | 75445 (0.00%) | 5541595159 (37.05%) |
| M11 | E45 | 11632637308 | 11375724049 (97.79%) | 10847463495 (93.25%) | 296373 (0.00%) | 4308684588 (37.04%) |
| A20 | E94 | 16846404764 | 16439018369 (97.58%) | 15607470976 (92.65%) | 369749 (0.00%) | 6231744451 (36.99%) |
| B37 | E128 | 15068300840 | 14663485032 (97.31%) | 13906783783 (92.29%) | 965115 (0.01%) | 5578078795 (37.02%) |
| E2 | E6 | 11830260572 | 11459205913 (96.86%) | 10831228898 (91.56%) | 101086 (0.00%) | 4382948341 (37.05%) |
| A41 | E117 | 16651721648 | 16051748486 (96.40%) | 15024186805 (90.23%) | 413390 (0.00%) | 6167787259 (37.04%) |
| M9 | E21 | 16387286448 | 16029477661 (97.82%) | 15311062063 (93.43%) | 82096 (0.00%) | 6064429032 (37.01%) |
| E14 | E31 | 12947497248 | 12571490923 (97.10%) | 11897221663 (91.89%) | 36487 (0.00%) | 4794905840 (37.03%) |
| A26 | E102 | 13159239004 | 12756137725 (96.94%) | 12060924337 (91.65%) | 1530958 (0.01%) | 4868269137 (37.00%) |
| B29 | E121 | 11459328008 | 11095637518 (96.83%) | 10459451431 (91.27%) | 614094 (0.01%) | 4237799805 (36.98%) |
| M17 | E49 | 15157378652 | 14616960489 (96.43%) | 13718989151 (90.51%) | 1692477 (0.01%) | 5610753679 (37.02%) |
| A3 | E75 | 15089729552 | 14633837607 (96.98%) | 13833837455 (91.68%) | 1797881 (0.01%) | 5580531990 (36.98%) |
| B39 | E129 | 14707689892 | 14210006213 (96.62%) | 13352574882 (90.79%) | 181468 (0.00%) | 5441516440 (37.00%) |
| B32 | E124 | 17002931656 | 16652341454 (97.94%) | 15912560883 (93.59%) | 185975 (0.00%) | 6287610492 (36.98%) |
| A27 | C30 | 14935793868 | 14581440448 (97.63%) | 13901421360 (93.07%) | 14070 (0.00%) | 5523574394 (36.98%) |
| H6 | E7 | 12776356340 | 12383425648 (96.92%) | 11703177203 (91.60%) | 93437 (0.00%) | 4746738955 (37.15%) |
| H3 | C1 | 16286757272 | 15889586996 (97.56%) | 15168538519 (93.13%) | 449389 (0.00%) | 6061456876 (37.22%) |
| E9 | E28 | 13938160100 | 13625501319 (97.76%) | 12985446881 (93.16%) | 162246 (0.00%) | 5156337004 (36.99%) |
| A14 | C25 | 14210024072 | 13798523260 (97.10%) | 13069801135 (91.98%) | 1705923 (0.01%) | 5254396102 (36.98%) |
| E3 | E8 | 13678071612 | 13280982261 (97.10%) | 12566775505 (91.88%) | 38755 (0.00%) | 5062028244 (37.01%) |
| A30 | E108 | 19618628236 | 19027538842 (96.99%) | 17951630100 (91.50%) | 1917174 (0.01%) | 7250826174 (36.96%) |
| E10 | B1 | 14826719020 | 14331910419 (96.66%) | 13482469101 (90.93%) | 150717 (0.00%) | 5490581890 (37.03%) |
| A29 | E107 | 14258436796 | 13873658345 (97.30%) | 13159883147 (92.30%) | 17970 (0.00%) | 5283348769 (37.05%) |
| M6 | C4 | 17259257140 | 16644146084 (96.44%) | 15628221122 (90.55%) | 784007 (0.00%) | 6387222185 (37.01%) |
| A25 | C29 | 12449803980 | 12032608498 (96.65%) | 11335187697 (91.05%) | 1483670 (0.01%) | 4599635791 (36.95%) |
| G1 | F4 | 10371313232 | 10098364606 (97.37%) | 9602188017 (92.58%) | 75449 (0.00%) | 3839106919 (37.02%) |
| A34 | C33 | 19109955476 | 18621335353 (97.44%) | 17675566872 (92.49%) | 1785905 (0.01%) | 7056190215 (36.92%) |
| E18 | E44 | 11954142740 | 11615985628 (97.17%) | 11021418791 (92.20%) | 42849 (0.00%) | 4423578205 (37.00%) |
| B4 | C22 | 16502308752 | 16048261879 (97.25%) | 15184597396 (92.01%) | 291246 (0.00%) | 6091064848 (36.91%) |
| M8 | E20 | 11393549460 | 11036151622 (96.86%) | 10424571480 (91.50%) | 125655 (0.00%) | 4213222357 (36.98%) |
| G5 | F9 | 11251969464 | 10792646884 (95.92%) | 10085048865 (89.63%) | 585475 (0.01%) | 4160952467 (36.98%) |
| A24 | E99 | 13093598864 | 12744038652 (97.33%) | 12100678691 (92.42%) | 11866 (0.00%) | 4829501154 (36.88%) |
| M18 | E50 | 17248560012 | 16791073743 (97.35%) | 15918564113 (92.29%) | 832316 (0.00%) | 6377174479 (36.97%) |
| B46 | E142 | 16204935660 | 15719529288 (97.00%) | 14849158143 (91.63%) | 329235 (0.00%) | 5983479415 (36.92%) |
| M14 | E47 | 13336182536 | 12853957088 (96.38%) | 12084540140 (90.61%) | 931065 (0.01%) | 4925208212 (36.93%) |
| A13 | E86 | 12445348060 | 12143851526 (97.58%) | 11558258070 (92.87%) | 11322 (0.00%) | 4589382396 (36.88%) |
| H2 | E2 | 15651760640 | 15265978499 (97.54%) | 14491747557 (92.59%) | 118292 (0.00%) | 5788691314 (36.98%) |
| A28 | C31 | 10356636144 | 10079616935 (97.33%) | 9568699510 (92.39%) | 125494 (0.00%) | 3820836841 (36.89%) |
| K3 | B9 | 11426745480 | 11120311488 (97.32%) | 10548048412 (92.31%) | 9886 (0.00%) | 4232369263 (37.04%) |
| A23 | C28 | 10108302116 | 9818658915 (97.13%) | 9292473915 (91.93%) | 43257 (0.00%) | 3726200179 (36.86%) |
| B45 | E141 | 13646061988 | 13221894633 (96.89%) | 12483334680 (91.48%) | 221487 (0.00%) | 5028979790 (36.85%) |
| B20 | E97 | 10352728308 | 10094576070 (97.51%) | 9583124608 (92.57%) | 31133 (0.00%) | 3812931699 (36.83%) |
| B22 | E100 | 14374952972 | 13893178301 (96.65%) | 13057703993 (90.84%) | 146675 (0.00%) | 5294990186 (36.83%) |
| A8 | E78 | 10007975004 | 9657007165 (96.49%) | 9043360399 (90.36%) | 129174 (0.00%) | 3689719254 (36.87%) |
| A17 | E91 | 11664611600 | 11383014180 (97.59%) | 10835195216 (92.89%) | 140185 (0.00%) | 4298676634 (36.85%) |
| A18 | E92 | 18838116324 | 18373779335 (97.54%) | 17466755222 (92.72%) | 23847 (0.00%) | 6941023664 (36.85%) |
| A19 | E93 | 10494640892 | 10132627114 (96.55%) | 9495105518 (90.48%) | 137951 (0.00%) | 3866918097 (36.85%) |
| M1 | C3 | 14068626284 | 13714558388 (97.48%) | 13048015714 (92.75%) | 227564 (0.00%) | 5180000848 (36.82%) |
| B28 | E118 | 13438320048 | 12994568594 (96.70%) | 12218814939 (90.93%) | 163167 (0.00%) | 4951251384 (36.84%) |
| M10 | E22 | 12084781788 | 11824907560 (97.85%) | 11300332679 (93.51%) | 339500 (0.00%) | 4449883482 (36.82%) |
| A21 | E95 | 12651844620 | 12290679481 (97.15%) | 11630335751 (91.93%) | 685320 (0.01%) | 4657865442 (36.82%) |
| A39 | E115 | 10344736560 | 10091027279 (97.55%) | 9601405485 (92.81%) | 124284 (0.00%) | 3810091352 (36.83%) |
| H18 | E43 | 11687483960 | 11239415238 (96.17%) | 10534417648 (90.13%) | 602682 (0.01%) | 4325203391 (37.01%) |
| B31 | E123 | 14266216552 | 13965882901 (97.89%) | 13356934073 (93.63%) | 102094 (0.00%) | 5247613387 (36.78%) |
| A33 | E110 | 17661812136 | 17157707577 (97.15%) | 16278678770 (92.17%) | 61805 (0.00%) | 6507106581 (36.84%) |
| E6 | E12 | 12050373676 | 11634803330 (96.55%) | 10959907013 (90.95%) | 609167 (0.01%) | 4445512705 (36.89%) |
| A22 | E98 | 17881621852 | 17355434511 (97.06%) | 16425179133 (91.86%) | 64534 (0.00%) | 6579552201 (36.80%) |
| A35 | E111 | 10987331032 | 10718677863 (97.55%) | 10199583298 (92.83%) | 10107 (0.00%) | 4039517453 (36.77%) |
| A10 | E81 | 14157816808 | 13752858946 (97.14%) | 13012078800 (91.91%) | 773310 (0.01%) | 5205226227 (36.77%) |
| H7 | A1 | 9927361980 | 9527865489 (95.98%) | 8919910896 (89.85%) | 515524 (0.01%) | 3689605790 (37.17%) |
| M28 | F16 | 10058010372 | 9654630491 (95.99%) | 9020952797 (89.69%) | 519845 (0.01%) | 3703170298 (36.82%) |
| G2 | F6 | 12966374172 | 12645591489 (97.53%) | 12038790136 (92.85%) | 11004 (0.00%) | 4787266704 (36.92%) |
| M25 | E53 | 12360229476 | 11950647987 (96.69%) | 11278555985 (91.25%) | 868873 (0.01%) | 4554074026 (36.84%) |
| G3 | F7 | 13261054148 | 12873789660 (97.08%) | 12185618435 (91.89%) | 38322 (0.00%) | 4876424934 (36.77%) |
| B11 | E72 | 14374923772 | 13898350689 (96.68%) | 13069231824 (90.92%) | 145348 (0.00%) | 5280520997 (36.73%) |
| M15 | A5 | 10793184612 | 10604969031 (98.26%) | 10196528261 (94.47%) | 48670 (0.00%) | 3962359800 (36.71%) |

**Supplementary Tables 3.** Statistics after sequencing data filtering.

| **ID** | **sample** | **CleanData(bp)** | **AF_Q20(%)** | **AF_Q30(%)** | **AF_N(%)** | **AF_GC(%)** |
| --- | --- | --- | --- | --- | --- | --- |
| C3 | E149 | 15367587092 | 14961396534 (97.36%) | 14192179449 (92.35%) | 132574 (0.00%) | 5795045206 (37.71%) |
| K4 | E35 | 13746584180 | 13496636584 (98.18%) | 12953617315 (94.23%) | 61543 (0.00%) | 5182296565 (37.70%) |
| B15 | E84 | 10595138892 | 10320845067 (97.41%) | 9788866158 (92.39%) | 12764 (0.00%) | 3991446809 (37.67%) |
| H9 | C2 | 14246196952 | 13969042243 (98.05%) | 13391740326 (94.00%) | 40706 (0.00%) | 5351419050 (37.56%) |
| E23 | C18 | 9929319668 | 9672736140 (97.42%) | 9183104914 (92.48%) | 49709 (0.00%) | 3728003619 (37.55%) |
| H17 | E41 | 12854167952 | 12466956370 (96.99%) | 11777215170 (91.62%) | 70269 (0.00%) | 4827092415 (37.55%) |
| F2 | E24 | 10296841576 | 10053198255 (97.63%) | 9563568152 (92.88%) | 30709 (0.00%) | 3861782812 (37.50%) |
| E24 | E59 | 11414647808 | 11099171678 (97.24%) | 10493559502 (91.93%) | 9788 (0.00%) | 4280432045 (37.50%) |
| F4 | E25 | 12722299628 | 12312925006 (96.78%) | 11590242849 (91.10%) | 70580 (0.00%) | 4770979263 (37.50%) |
| E25 | E60 | 11699057544 | 11361253043 (97.11%) | 10746361558 (91.86%) | 25468 (0.00%) | 4384866702 (37.48%) |
| E28 | E61 | 12620670900 | 12157826252 (96.33%) | 11406875268 (90.38%) | 47406 (0.00%) | 4730110316 (37.48%) |
| C6 | E152 | 15422639128 | 14968972212 (97.06%) | 14158505622 (91.80%) | 84380 (0.00%) | 5779247760 (37.47%) |
| H15 | E38 | 12555579616 | 12233632290 (97.44%) | 11600936936 (92.40%) | 10599 (0.00%) | 4703799133 (37.46%) |
| F7 | B6 | 11139093540 | 10858148897 (97.48%) | 10307001777 (92.53%) | 9552 (0.00%) | 4171338251 (37.45%) |
| C5 | E151 | 14759512776 | 14309696177 (96.95%) | 13506869056 (91.51%) | 32227 (0.00%) | 5528039540 (37.45%) |
| K8 | E56 | 10275788300 | 9947734552 (96.81%) | 9384785333 (91.33%) | 111244 (0.00%) | 3848190414 (37.45%) |
| E27 | B13 | 9406008536 | 9123596688 (97.00%) | 8604395162 (91.48%) | 27774 (0.00%) | 3521334386 (37.44%) |
| E17 | E42 | 9512367220 | 9186381278 (96.57%) | 8642738224 (90.86%) | 35501 (0.00%) | 3560054535 (37.43%) |
| F10 | E33 | 10866230976 | 10448611062 (96.16%) | 9780043133 (90.00%) | 44399 (0.00%) | 4066807405 (37.43%) |
| E19 | E55 | 10429896728 | 10186922575 (97.67%) | 9690863233 (92.91%) | 31539 (0.00%) | 3903007620 (37.42%) |
| E20 | E57 | 12156401420 | 11901920083 (97.91%) | 11350967786 (93.37%) | 141999 (0.00%) | 4547914311 (37.41%) |
| C8 | E154 | 13250976892 | 12765911631 (96.34%) | 11980193764 (90.41%) | 49266 (0.00%) | 4957756193 (37.41%) |
| K7 | E39 | 13688494092 | 13318829220 (97.30%) | 12611083075 (92.13%) | 49935 (0.00%) | 5119267578 (37.40%) |
| K5 | B10 | 12799757724 | 12488078963 (97.56%) | 11887619406 (92.87%) | 115879 (0.00%) | 4784709794 (37.38%) |
| C2 | E148 | 11866487968 | 11526401597 (97.13%) | 10909286476 (91.93%) | 191398 (0.00%) | 4433963391 (37.37%) |
| E22 | C17 | 11554119804 | 11212576762 (97.04%) | 10593953392 (91.69%) | 185869 (0.00%) | 4318213354 (37.37%) |
| K2 | B8 | 12933423460 | 12639335502 (97.73%) | 12055165271 (93.21%) | 263345 (0.00%) | 4831933095 (37.36%) |
| A1 | E69 | 11404949204 | 11102614713 (97.35%) | 10522093012 (92.26%) | 46635 (0.00%) | 4260210958 (37.35%) |
| C7 | E153 | 11398787128 | 11051957410 (96.96%) | 10421083869 (91.42%) | 33881 (0.00%) | 4257278808 (37.35%) |
| C1 | E147 | 12822390184 | 12414925657 (96.82%) | 11695501908 (91.21%) | 72121 (0.00%) | 4788907788 (37.35%) |
| K9 | E63 | 11911127544 | 11685763586 (98.11%) | 11189618831 (93.94%) | 73806 (0.00%) | 4447429523 (37.34%) |
| B48 | E145 | 14685249716 | 14388780261 (97.98%) | 13763682662 (93.72%) | 102857 (0.00%) | 5482884141 (37.34%) |
| F3 | C6 | 13678357688 | 13394473100 (97.92%) | 12783599517 (93.46%) | 161048 (0.00%) | 5107953640 (37.34%) |
| F5 | E30 | 13384410072 | 13017949944 (97.26%) | 12342069211 (92.21%) | 211786 (0.00%) | 4998402058 (37.34%) |
| A2 | E74 | 16487326748 | 16010781197 (97.11%) | 15127477576 (91.75%) | 152471 (0.00%) | 6157102585 (37.34%) |
| G4 | F8 | 13145556152 | 12752458405 (97.01%) | 12050412818 (91.67%) | 71246 (0.00%) | 4908829271 (37.34%) |
| C4 | E150 | 11266501196 | 10908004585 (96.82%) | 10299796335 (91.42%) | 83438 (0.00%) | 4207265539 (37.34%) |
| F11 | A2 | 11881134828 | 11477362732 (96.60%) | 10809499670 (90.98%) | 45414 (0.00%) | 4435873819 (37.34%) |
| B16 | E87 | 19096024208 | 18410214763 (96.41%) | 17225449987 (90.20%) | 142934 (0.00%) | 7130814829 (37.34%) |
| M5 | E17 | 13960851204 | 13476778023 (96.53%) | 12675858721 (90.80%) | 129637 (0.00%) | 5211006479 (37.33%) |
| M4 | E16 | 11707522488 | 11497892961 (98.21%) | 11030744366 (94.22%) | 52889 (0.00%) | 4369393520 (37.32%) |
| E21 | E58 | 12734579092 | 12325523216 (96.79%) | 11603825039 (91.12%) | 72031 (0.00%) | 4753043669 (37.32%) |
| H13 | B2 | 14485476252 | 14182112062 (97.91%) | 13513887686 (93.29%) | 109480 (0.00%) | 5404799045 (37.31%) |
| A5 | E77 | 12005732788 | 11713934825 (97.57%) | 11136974980 (92.76%) | 11162 (0.00%) | 4478916291 (37.31%) |
| K6 | E36 | 9705689684 | 9325467549 (96.08%) | 8719029688 (89.83%) | 39343 (0.00%) | 3621329139 (37.31%) |
| M2 | E14 | 14090748552 | 13760595892 (97.66%) | 13109057785 (93.03%) | 222040 (0.00%) | 5255178115 (37.30%) |
| F6 | B4 | 11176834596 | 10898738115 (97.51%) | 10366114144 (92.75%) | 56489 (0.00%) | 4168555344 (37.30%) |
| B49 | E146 | 16492974800 | 16183210019 (98.12%) | 15489778098 (93.92%) | 180594 (0.00%) | 6150038920 (37.29%) |
| B19 | E96 | 13586581760 | 13203768343 (97.18%) | 12507233908 (92.06%) | 48656 (0.00%) | 5066564078 (37.29%) |
| B23 | E101 | 15967551088 | 15522150650 (97.21%) | 14675257448 (91.91%) | 243751 (0.00%) | 5954743498 (37.29%) |
| A45 | E132 | 12485038208 | 12129445820 (97.15%) | 11453116597 (91.73%) | 36940 (0.00%) | 4656170665 (37.29%) |
| H14 | E37 | 14638242636 | 14304361976 (97.72%) | 13642967976 (93.20%) | 297758 (0.00%) | 5456637849 (37.28%) |
| G9 | F13 | 14593243900 | 14222902566 (97.46%) | 13514901336 (92.61%) | 74540 (0.00%) | 5440785356 (37.28%) |
| H10 | C7 | 11777024416 | 11558709447 (98.15%) | 11076495263 (94.05%) | 73152 (0.00%) | 4389686044 (37.27%) |
| F15 | C15 | 12551069172 | 12287332533 (97.90%) | 11721624385 (93.39%) | 146541 (0.00%) | 4677550702 (37.27%) |
| B13 | E79 | 13180074564 | 12858293171 (97.56%) | 12224017037 (92.75%) | 12510 (0.00%) | 4912689821 (37.27%) |
| B42 | E139 | 11890494516 | 11646382698 (97.95%) | 11135450316 (93.65%) | 84033 (0.00%) | 4430026387 (37.26%) |
| E26 | C19 | 16439063300 | 15944153697 (96.99%) | 15056173418 (91.59%) | 88367 (0.00%) | 6125757039 (37.26%) |
| H11 | E26 | 15392261584 | 15021818520 (97.59%) | 14298389072 (92.89%) | 77412 (0.00%) | 5733059251 (37.25%) |
| F1 | E23 | 14631680992 | 14233086141 (97.28%) | 13491018504 (92.20%) | 54072 (0.00%) | 5449944959 (37.25%) |
| K10 | F3 | 10733131916 | 10515039998 (97.97%) | 10060083490 (93.73%) | 218113 (0.00%) | 3997082357 (37.24%) |
| B24 | E103 | 14567068712 | 14193493700 (97.44%) | 13472200731 (92.48%) | 13418 (0.00%) | 5425384175 (37.24%) |
| E15 | E34 | 15816068596 | 15372788227 (97.20%) | 14552380805 (92.01%) | 86344 (0.00%) | 5889746655 (37.24%) |
| B27 | E106 | 16178750720 | 15633971375 (96.63%) | 14663204394 (90.63%) | 118606 (0.00%) | 6024207466 (37.24%) |
| H8 | E11 | 11308255788 | 10902997325 (96.42%) | 10242189403 (90.57%) | 42378 (0.00%) | 4210694670 (37.24%) |
| A47 | E134 | 14682335524 | 14063598217 (95.79%) | 13073662579 (89.04%) | 80211 (0.00%) | 5467108608 (37.24%) |
| B14 | E82 | 10908497620 | 10630557924 (97.45%) | 10091987563 (92.51%) | 130507 (0.00%) | 4061009045 (37.23%) |
| H4 | E4 | 12056130076 | 11743428656 (97.41%) | 11136870808 (92.38%) | 10521 (0.00%) | 4488988989 (37.23%) |
| A49 | E135 | 13856447880 | 13497565256 (97.41%) | 12806583797 (92.42%) | 12853 (0.00%) | 5157786720 (37.22%) |
| C9 | E155 | 11051898784 | 10736227866 (97.14%) | 10167896608 (92.00%) | 40367 (0.00%) | 4113955330 (37.22%) |
| B38 | C35 | 13117578356 | 12629263591 (96.28%) | 11805175147 (90.00%) | 91293 (0.00%) | 4881784410 (37.22%) |
| H23 | F1 | 17724564612 | 17332638719 (97.79%) | 16489444666 (93.03%) | 134488 (0.00%) | 6595695695 (37.21%) |
| B18 | C26 | 12025620372 | 11658619522 (96.95%) | 10994338581 (91.42%) | 52970 (0.00%) | 4474756611 (37.21%) |
| F13 | E54 | 11100423036 | 10849150393 (97.74%) | 10336185846 (93.12%) | 32856 (0.00%) | 4128896394 (37.20%) |
| M22 | C13 | 10777347568 | 10526815582 (97.68%) | 10009107576 (92.87%) | 183955 (0.00%) | 4009112875 (37.20%) |
| F8 | B7 | 12175827004 | 11826566434 (97.13%) | 11191670956 (91.92%) | 27609 (0.00%) | 4529752508 (37.20%) |
| B35 | E127 | 11723931284 | 11377587975 (97.05%) | 10756896090 (91.75%) | 57611 (0.00%) | 4361478188 (37.20%) |
| G7 | F11 | 17004136340 | 16685298209 (98.12%) | 16011962061 (94.17%) | 48580 (0.00%) | 6324281308 (37.19%) |
| B41 | E130 | 14076020340 | 13656948266 (97.02%) | 12918253099 (91.77%) | 50476 (0.00%) | 5235220058 (37.19%) |
| B9 | E70 | 16322453916 | 15850482200 (97.11%) | 14959241083 (91.65%) | 154665 (0.00%) | 6070230827 (37.19%) |
| H21 | A7 | 13274485252 | 13022826074 (98.10%) | 12475475638 (93.98%) | 81314 (0.00%) | 4935145026 (37.18%) |
| B25 | E104 | 11100583828 | 10823495137 (97.50%) | 10280977962 (92.62%) | 131798 (0.00%) | 4127324892 (37.18%) |
| G6 | F10 | 11570288336 | 11177117480 (96.60%) | 10520035744 (90.92%) | 43689 (0.00%) | 4301636440 (37.18%) |
| M26 | F14 | 13235332696 | 12997806682 (98.21%) | 12466128839 (94.19%) | 59741 (0.00%) | 4919158159 (37.17%) |
| F12 | A3 | 15102960652 | 14800037196 (97.99%) | 14179475576 (93.89%) | 43779 (0.00%) | 5613539026 (37.17%) |
| B2 | C21 | 11984646692 | 11723088133 (97.82%) | 11185227425 (93.33%) | 11043 (0.00%) | 4454143645 (37.17%) |
| M24 | E52 | 12772457984 | 12480683049 (97.72%) | 11898564690 (93.16%) | 200607 (0.00%) | 4747649957 (37.17%) |
| A42 | E119 | 15497967508 | 15062876532 (97.19%) | 14212427099 (91.71%) | 337564 (0.00%) | 5760381944 (37.17%) |
| M3 | E15 | 11264579484 | 10887477470 (96.65%) | 10254203993 (91.03%) | 138762 (0.00%) | 4186897756 (37.17%) |
| H19 | B11 | 10883266852 | 10511504899 (96.58%) | 9890683054 (90.88%) | 40945 (0.00%) | 4045146658 (37.17%) |
| H16 | E40 | 12617250488 | 12266882617 (97.22%) | 11620681722 (92.10%) | 27552 (0.00%) | 4688292618 (37.16%) |
| A44 | E131 | 18945481076 | 18337595962 (96.79%) | 17279394340 (91.21%) | 67708 (0.00%) | 7041049532 (37.16%) |
| A37 | E113 | 12170970984 | 11773019515 (96.73%) | 11084876345 (91.08%) | 805766 (0.01%) | 4522855094 (37.16%) |
| F14 | B12 | 12031752212 | 11642892612 (96.77%) | 10957777226 (91.07%) | 66187 (0.00%) | 4470479402 (37.16%) |
| A16 | E90 | 15224335796 | 14867769147 (97.66%) | 14156714816 (92.99%) | 14345 (0.00%) | 5656131858 (37.15%) |
| B10 | E71 | 14023497176 | 13687715862 (97.61%) | 12995937462 (92.67%) | 83954 (0.00%) | 5210028189 (37.15%) |
| H5 | E5 | 12615487816 | 12234509551 (96.98%) | 11551142220 (91.56%) | 27483 (0.00%) | 4686276551 (37.15%) |
| E1 | E3 | 19114946600 | 18536609462 (96.97%) | 17485889103 (91.48%) | 128197 (0.00%) | 7101126853 (37.15%) |
| B17 | E88 | 14092528764 | 13570088764 (96.29%) | 12671906175 (89.92%) | 42453 (0.00%) | 5235622781 (37.15%) |
| H20 | C16 | 17198591092 | 16868016892 (98.08%) | 16177147410 (94.06%) | 49947 (0.00%) | 6387390446 (37.14%) |
| H1 | E1 | 16672034944 | 16299139682 (97.76%) | 15503048815 (92.99%) | 125737 (0.00%) | 6192786151 (37.14%) |
| C11 | E157 | 15275789464 | 14993894032 (98.15%) | 14349092125 (93.93%) | 167914 (0.00%) | 5671576817 (37.13%) |
| H12 | E27 | 16375842832 | 16027123859 (97.87%) | 15262054299 (93.20%) | 124497 (0.00%) | 6080914229 (37.13%) |
| B7 | E67 | 13214230472 | 12813117107 (96.96%) | 12083039516 (91.44%) | 59078 (0.00%) | 4906709765 (37.13%) |
| B1 | C20 | 11782486100 | 11449525253 (97.17%) | 10823984568 (91.87%) | 189916 (0.00%) | 4373416557 (37.12%) |
| B8 | E68 | 13255226340 | 12870338442 (97.10%) | 12176282298 (91.86%) | 47671 (0.00%) | 4919906722 (37.12%) |
| E8 | C8 | 10804464632 | 10553322017 (97.68%) | 10039409534 (92.92%) | 32672 (0.00%) | 4009667623 (37.11%) |
| M19 | E51 | 9840752280 | 9515883748 (96.70%) | 8958951247 (91.04%) | 107670 (0.00%) | 3652367173 (37.11%) |
| A7 | C24 | 16981902872 | 16564087241 (97.54%) | 15733160652 (92.65%) | 19928 (0.00%) | 6300040556 (37.10%) |
| B40 | C36 | 11198843976 | 10855954599 (96.94%) | 10244956897 (91.48%) | 49691 (0.00%) | 4155007567 (37.10%) |
| B12 | E73 | 16402473604 | 15875961234 (96.79%) | 14929035010 (91.02%) | 280673 (0.00%) | 6085444010 (37.10%) |
| A53 | E143 | 14771542944 | 14279747032 (96.67%) | 13409661645 (90.78%) | 82731 (0.00%) | 5479762461 (37.10%) |
| M13 | E46 | 12144379384 | 11868719126 (97.73%) | 11316591318 (93.18%) | 194572 (0.00%) | 4504919760 (37.09%) |
| M16 | A6 | 15233905240 | 14880520988 (97.68%) | 14154741868 (92.92%) | 98107 (0.00%) | 5650948547 (37.09%) |
| B3 | E64 | 10541865540 | 10292649525 (97.64%) | 9793757103 (92.90%) | 125621 (0.00%) | 3910211035 (37.09%) |
| B26 | E105 | 18129141944 | 17689057654 (97.57%) | 16806811222 (92.71%) | 149882 (0.00%) | 6724534240 (37.09%) |
| E12 | E29 | 10930546460 | 10654106186 (97.47%) | 10118406559 (92.57%) | 55862 (0.00%) | 4053890723 (37.09%) |
| F16 | F2 | 13134264440 | 12781256978 (97.31%) | 12122070171 (92.29%) | 210861 (0.00%) | 4871448799 (37.09%) |
| K1 | E18 | 15814748524 | 15480704638 (97.89%) | 14748150075 (93.26%) | 119779 (0.00%) | 5864522586 (37.08%) |
| A9 | E80 | 17569313084 | 17101557004 (97.34%) | 16165449441 (92.01%) | 381614 (0.00%) | 6514646269 (37.08%) |
| A15 | E89 | 12930540196 | 12563318760 (97.16%) | 11893034793 (91.98%) | 858485 (0.01%) | 4794711115 (37.08%) |
| A38 | E114 | 15232470452 | 14864047160 (97.58%) | 14137806240 (92.81%) | 14579 (0.00%) | 5646498871 (37.07%) |
| E13 | B5 | 11453556724 | 11163713688 (97.47%) | 10587723535 (92.44%) | 9919 (0.00%) | 4245229934 (37.06%) |
| B30 | E122 | 13193015808 | 12806065034 (97.07%) | 12109447116 (91.79%) | 47253 (0.00%) | 4889720024 (37.06%) |
| B34 | E126 | 11079298148 | 10752084233 (97.05%) | 10160077918 (91.70%) | 177237 (0.00%) | 4105929266 (37.06%) |
| B43 | C38 | 15867818992 | 15538876076 (97.93%) | 14827666036 (93.44%) | 173642 (0.00%) | 5879650412 (37.05%) |
| M23 | C14 | 13553997660 | 13235190569 (97.65%) | 12608200253 (93.02%) | 214027 (0.00%) | 5022125325 (37.05%) |
| A46 | E133 | 12999192024 | 12652693369 (97.33%) | 11961224088 (92.02%) | 78392 (0.00%) | 4815857101 (37.05%) |
| E4 | E9 | 15721915756 | 15255257388 (97.03%) | 14410125262 (91.66%) | 86181 (0.00%) | 5825634064 (37.05%) |
| A48 | C37 | 13459735296 | 13044376873 (96.91%) | 12299381673 (91.38%) | 216475 (0.00%) | 4986253813 (37.05%) |
| C12 | E158 | 12156089164 | 11766406306 (96.79%) | 11068338805 (91.05%) | 66882 (0.00%) | 4503414615 (37.05%) |
| B44 | E140 | 12710440904 | 12232186745 (96.24%) | 11438243960 (89.99%) | 70183 (0.00%) | 4708983362 (37.05%) |
| N3 | E48 | 15144393764 | 14796881523 (97.71%) | 14106562165 (93.15%) | 77166 (0.00%) | 5608940813 (37.04%) |
| A4 | E76 | 10757215864 | 10435828867 (97.01%) | 9861153675 (91.67%) | 714259 (0.01%) | 3984932020 (37.04%) |
| B36 | C34 | 10426668204 | 10088009768 (96.75%) | 9519072942 (91.30%) | 79204 (0.00%) | 3862294165 (37.04%) |
| E5 | E10 | 9956551012 | 9636764175 (96.79%) | 9056551597 (90.96%) | 29257 (0.00%) | 3688311548 (37.04%) |
| B6 | E66 | 14796706084 | 14301849583 (96.66%) | 13416990285 (90.68%) | 44295 (0.00%) | 5481012933 (37.04%) |
| A6 | C23 | 10612958136 | 10332028608 (97.35%) | 9798150588 (92.32%) | 125890 (0.00%) | 3929526727 (37.03%) |
| F9 | E32 | 12305537708 | 11943934098 (97.06%) | 11292109742 (91.76%) | 66914 (0.00%) | 4556586988 (37.03%) |
| A43 | E120 | 11728440444 | 11356332344 (96.83%) | 10692789945 (91.17%) | 51960 (0.00%) | 4343521226 (37.03%) |
| B21 | C27 | 11964097344 | 11669562056 (97.54%) | 11069497961 (92.52%) | 71336 (0.00%) | 4429075694 (37.02%) |
| N1 | C5 | 14177051772 | 13821234724 (97.49%) | 13112681580 (92.49%) | 53163 (0.00%) | 5248346071 (37.02%) |
| M21 | C12 | 12046438768 | 11774006939 (97.74%) | 11227514893 (93.20%) | 245364 (0.00%) | 4458364422 (37.01%) |
| M12 | C10 | 12243741656 | 11960208801 (97.68%) | 11397310117 (93.09%) | 193592 (0.00%) | 4531405013 (37.01%) |
| H22 | E62 | 12911859364 | 12609419767 (97.66%) | 12011517211 (93.03%) | 65998 (0.00%) | 4778919611 (37.01%) |
| A52 | E138 | 14023468764 | 13701493258 (97.70%) | 13026420600 (92.89%) | 84491 (0.00%) | 5189399617 (37.01%) |
| M7 | E19 | 12515754612 | 12195398964 (97.44%) | 11559266022 (92.36%) | 47028 (0.00%) | 4631582403 (37.01%) |
| A31 | C32 | 15628682736 | 15213732837 (97.34%) | 14384489443 (92.04%) | 339318 (0.00%) | 5784006075 (37.01%) |
| C13 | E159 | 12200250348 | 11852797984 (97.15%) | 11208600449 (91.87%) | 194852 (0.00%) | 4514938617 (37.01%) |
| N2 | C9 | 13650751592 | 13231667247 (96.93%) | 12494365783 (91.53%) | 147915 (0.00%) | 5051656181 (37.01%) |
| B33 | E125 | 12918592772 | 12479270587 (96.60%) | 11717409573 (90.70%) | 71772 (0.00%) | 4781631977 (37.01%) |
| C10 | E156 | 10910407644 | 10701221906 (98.08%) | 10247984121 (93.93%) | 77068 (0.00%) | 4036706678 (37.00%) |
| K11 | F5 | 10393943616 | 10165991487 (97.81%) | 9689600460 (93.22%) | 181325 (0.00%) | 3845271000 (37.00%) |
| M27 | F15 | 11483636436 | 11206259591 (97.58%) | 10658070095 (92.81%) | 102909 (0.00%) | 4248809248 (37.00%) |
| A51 | E137 | 10392507376 | 10145023293 (97.62%) | 9641635668 (92.77%) | 30498 (0.00%) | 3845715166 (37.00%) |
| A40 | E116 | 16321904788 | 15899125428 (97.41%) | 15074697474 (92.36%) | 19344 (0.00%) | 6039119815 (37.00%) |
| A12 | E85 | 10889207504 | 10602362839 (97.37%) | 10051742275 (92.31%) | 45495 (0.00%) | 4029175869 (37.00%) |
| E7 | E13 | 11556717088 | 11243079994 (97.29%) | 10666139756 (92.29%) | 41358 (0.00%) | 4276216971 (37.00%) |
| E16 | A4 | 10780864940 | 10474412939 (97.16%) | 9893773324 (91.77%) | 32022 (0.00%) | 3988666751 (37.00%) |
| A32 | E109 | 12263952240 | 11875924119 (96.84%) | 11189284508 (91.24%) | 54817 (0.00%) | 4538274453 (37.00%) |
| B5 | E65 | 15235783068 | 14773166546 (96.96%) | 13908882711 (91.29%) | 146825 (0.00%) | 5635763232 (36.99%) |
| E11 | B3 | 12708964716 | 12367389475 (97.31%) | 11721148646 (92.23%) | 203958 (0.00%) | 4700356136 (36.98%) |
| A50 | E136 | 13407202096 | 13018425767 (97.10%) | 12307226924 (91.80%) | 159166 (0.00%) | 4958036386 (36.98%) |
| A36 | E112 | 11487142588 | 11142060389 (97.00%) | 10525962984 (91.63%) | 756572 (0.01%) | 4248294617 (36.98%) |
| B47 | E144 | 10540931300 | 10196759758 (96.73%) | 9621212130 (91.27%) | 78575 (0.00%) | 3898535964 (36.98%) |
| G8 | F12 | 13429727260 | 13157994238 (97.98%) | 12582895387 (93.69%) | 82647 (0.00%) | 4965409367 (36.97%) |
| A11 | E83 | 14508907296 | 14122813381 (97.34%) | 13395050793 (92.32%) | 51441 (0.00%) | 5363924668 (36.97%) |
| M20 | C11 | 14515187136 | 14195642147 (97.80%) | 13549830992 (93.35%) | 73000 (0.00%) | 5364371555 (36.96%) |
| M11 | E45 | 11288909400 | 11049039808 (97.88%) | 10537017487 (93.34%) | 193709 (0.00%) | 4171194506 (36.95%) |
| A20 | E94 | 16733220192 | 16339301690 (97.65%) | 15514826828 (92.72%) | 365861 (0.00%) | 6183438203 (36.95%) |
| B37 | E128 | 14853245764 | 14469026621 (97.41%) | 13725668630 (92.41%) | 89182 (0.00%) | 5488903530 (36.95%) |
| E2 | E6 | 11367508984 | 11026159955 (97.00%) | 10425707420 (91.71%) | 84499 (0.00%) | 4199906932 (36.95%) |
| A41 | E117 | 16472138292 | 15895401594 (96.50%) | 14881364025 (90.34%) | 197941 (0.00%) | 6087256751 (36.95%) |
| M9 | E21 | 15980963884 | 15641167421 (97.87%) | 14941117448 (93.49%) | 80214 (0.00%) | 5904013666 (36.94%) |
| E14 | E31 | 12628798276 | 12276389573 (97.21%) | 11620199924 (92.01%) | 26989 (0.00%) | 4665619574 (36.94%) |
| A26 | E102 | 13018174436 | 12635021041 (97.06%) | 11950241913 (91.80%) | 861624 (0.01%) | 4808729207 (36.94%) |
| B29 | E121 | 11345786728 | 11000152596 (96.95%) | 10372454502 (91.42%) | 50031 (0.00%) | 4190629641 (36.94%) |
| M17 | E49 | 14897037372 | 14384086828 (96.56%) | 13503881477 (90.65%) | 134730 (0.00%) | 5503606625 (36.94%) |
| A3 | E75 | 14963003732 | 14529479134 (97.10%) | 13739994043 (91.83%) | 988969 (0.01%) | 5525377521 (36.93%) |
| B39 | E129 | 14530698980 | 14055636239 (96.73%) | 13210772266 (90.92%) | 44640 (0.00%) | 5366337675 (36.93%) |
| B32 | E124 | 16744612808 | 16412288542 (98.02%) | 15686216071 (93.68%) | 183138 (0.00%) | 6182043203 (36.92%) |
| A27 | C30 | 14726084892 | 14390319859 (97.72%) | 13722145917 (93.18%) | 13746 (0.00%) | 5436652545 (36.92%) |
| H6 | E7 | 12378202612 | 12016091731 (97.07%) | 11360045343 (91.77%) | 67118 (0.00%) | 4570136575 (36.92%) |
| H3 | C1 | 15502116004 | 15157832336 (97.78%) | 14478458716 (93.40%) | 314702 (0.00%) | 5721553738 (36.91%) |
| E9 | E28 | 13487309404 | 13198385349 (97.86%) | 12581242131 (93.28%) | 157041 (0.00%) | 4978119170 (36.91%) |
| A14 | C25 | 14050158348 | 13659606306 (97.22%) | 12942271475 (92.11%) | 929859 (0.01%) | 5186439809 (36.91%) |
| E3 | E8 | 13234850184 | 12866723611 (97.22%) | 12177032513 (92.01%) | 28270 (0.00%) | 4884411165 (36.91%) |
| A30 | E108 | 19440854092 | 18873395667 (97.08%) | 17810238768 (91.61%) | 219372 (0.00%) | 7175550250 (36.91%) |
| E10 | B1 | 14309349096 | 13852544381 (96.81%) | 13036239623 (91.10%) | 80492 (0.00%) | 5281622138 (36.91%) |
| A29 | E107 | 14108613312 | 13744222703 (97.42%) | 13041054534 (92.43%) | 16863 (0.00%) | 5205645595 (36.90%) |
| M6 | C4 | 16682942348 | 16111480994 (96.57%) | 15131024962 (90.70%) | 194417 (0.00%) | 6155355989 (36.90%) |
| A25 | C29 | 12349543380 | 11952599166 (96.79%) | 11264366664 (91.21%) | 815408 (0.01%) | 4556241259 (36.89%) |
| G1 | F4 | 9930436528 | 9684602356 (97.52%) | 9212659710 (92.77%) | 48940 (0.00%) | 3662121222 (36.88%) |
| A34 | C33 | 18924485460 | 18454523255 (97.52%) | 17520363127 (92.58%) | 190568 (0.00%) | 6980209346 (36.88%) |
| E18 | E44 | 11484461160 | 11177381405 (97.33%) | 10608821466 (92.38%) | 41198 (0.00%) | 4234258938 (36.87%) |
| B4 | C22 | 16400575704 | 15959052185 (97.31%) | 15102517982 (92.09%) | 142099 (0.00%) | 6047203001 (36.87%) |
| M8 | E20 | 11082937268 | 10748348240 (96.98%) | 10155524246 (91.63%) | 120364 (0.00%) | 4086676013 (36.87%) |
| G5 | F9 | 10943574772 | 10513634366 (96.07%) | 9826853082 (89.80%) | 45048 (0.00%) | 4035339462 (36.87%) |
| A24 | E99 | 13029222428 | 12690076705 (97.40%) | 12051805441 (92.50%) | 11814 (0.00%) | 4802293095 (36.86%) |
| M18 | E50 | 16653401516 | 16229711643 (97.46%) | 15387357244 (92.40%) | 166248 (0.00%) | 6138203500 (36.86%) |
| B46 | E142 | 15986651208 | 15522970911 (97.10%) | 14666875517 (91.74%) | 167537 (0.00%) | 5891632542 (36.85%) |
| M14 | E47 | 13055977060 | 12607282953 (96.56%) | 11857820817 (90.82%) | 160388 (0.00%) | 4810573518 (36.85%) |
| A13 | E86 | 12368696076 | 12076666881 (97.64%) | 11496217570 (92.95%) | 11240 (0.00%) | 4557040480 (36.84%) |
| H2 | E2 | 15328078312 | 14971264480 (97.67%) | 14217291346 (92.75%) | 115790 (0.00%) | 5647459582 (36.84%) |
| A28 | C31 | 10284368108 | 10016778153 (97.40%) | 9511219313 (92.48%) | 122328 (0.00%) | 3788980519 (36.84%) |
| K3 | B9 | 11076828536 | 10795397056 (97.46%) | 10242881110 (92.47%) | 9577 (0.00%) | 4081138603 (36.84%) |
| A23 | C28 | 10030572856 | 9749020076 (97.19%) | 9228076024 (92.00%) | 41671 (0.00%) | 3694137384 (36.83%) |
| B45 | E141 | 13513920752 | 13105609456 (96.98%) | 12376029405 (91.58%) | 219444 (0.00%) | 4975637196 (36.82%) |
| B20 | E97 | 10319394972 | 10066301821 (97.55%) | 9557355899 (92.62%) | 31021 (0.00%) | 3798515935 (36.81%) |
| B22 | E100 | 14251004872 | 13789463265 (96.76%) | 12964346358 (90.97%) | 79271 (0.00%) | 5245437276 (36.81%) |
| A8 | E78 | 9932174108 | 9593257183 (96.59%) | 8985711939 (90.47%) | 66570 (0.00%) | 3656103046 (36.81%) |
| A17 | E91 | 11537127864 | 11266338633 (97.65%) | 10726250725 (92.97%) | 136639 (0.00%) | 4245364273 (36.80%) |
| A18 | E92 | 18638189216 | 18191383880 (97.60%) | 17296007205 (92.80%) | 22088 (0.00%) | 6858287025 (36.80%) |
| A19 | E93 | 10421943456 | 10071425384 (96.64%) | 9439695691 (90.58%) | 68733 (0.00%) | 3835285208 (36.80%) |
| M1 | C3 | 13818581588 | 13482265568 (97.57%) | 12829268629 (92.84%) | 218722 (0.00%) | 5082004466 (36.78%) |
| B28 | E118 | 13261570824 | 12838816821 (96.81%) | 12074910615 (91.05%) | 40060 (0.00%) | 4877816461 (36.78%) |
| M10 | E22 | 11833049552 | 11586356600 (97.92%) | 11073668199 (93.58%) | 240693 (0.00%) | 4350550905 (36.77%) |
| A21 | E95 | 12503262308 | 12157550478 (97.24%) | 11506248658 (92.03%) | 55546 (0.00%) | 4596952741 (36.77%) |
| A39 | E115 | 10199493648 | 9956955669 (97.62%) | 9475922043 (92.91%) | 121268 (0.00%) | 3749642458 (36.76%) |
| H18 | E43 | 11157132900 | 10748917654 (96.34%) | 10076828925 (90.32%) | 45365 (0.00%) | 4101756313 (36.76%) |
| B31 | E123 | 14133064572 | 13845632598 (97.97%) | 13244363603 (93.71%) | 99825 (0.00%) | 5194126937 (36.75%) |
| A33 | E110 | 17289751732 | 16819365178 (97.28%) | 15963346237 (92.33%) | 60445 (0.00%) | 6353656961 (36.75%) |
| E6 | E12 | 11562605492 | 11184510379 (96.73%) | 10539142741 (91.15%) | 43492 (0.00%) | 4249029551 (36.75%) |
| A22 | E98 | 17725819844 | 17225682823 (97.18%) | 16308338349 (92.00%) | 63991 (0.00%) | 6512017687 (36.74%) |
| A35 | E111 | 10907177816 | 10647278430 (97.62%) | 10133321611 (92.91%) | 10035 (0.00%) | 4006439543 (36.73%) |
| A10 | E81 | 14012509320 | 13625132264 (97.24%) | 12893795767 (92.02%) | 62315 (0.00%) | 5145042728 (36.72%) |
| H7 | A1 | 9380866444 | 9024896871 (96.21%) | 8452096415 (90.10%) | 37592 (0.00%) | 3444535896 (36.72%) |
| M28 | F16 | 9755402672 | 9376734733 (96.12%) | 8762252660 (89.82%) | 39847 (0.00%) | 3581513856 (36.71%) |
| G2 | F6 | 12173304804 | 11891836615 (97.69%) | 11324815655 (93.03%) | 10356 (0.00%) | 4468031439 (36.70%) |
| M25 | E53 | 11848661900 | 11476168339 (96.86%) | 10834051405 (91.44%) | 147116 (0.00%) | 4348933106 (36.70%) |
| G3 | F7 | 12978407744 | 12616151939 (97.21%) | 11945569413 (92.04%) | 28559 (0.00%) | 4761663363 (36.69%) |
| B11 | E72 | 14150681368 | 13695871833 (96.79%) | 12881936731 (91.03%) | 78693 (0.00%) | 5189487443 (36.67%) |
| M15 | A5 | 10385533388 | 10215776264 (98.37%) | 9824847081 (94.60%) | 46841 (0.00%) | 3802029655 (36.61%) |

**Supplementary Tables 4.** Statistics on the depth of sequencing of high quality SNP markers in each sample.

| **ID** | depth | **ID** | depth | **ID** | depth | **ID** | depth | **ID** | depth |
| --- | --- | --- | --- | --- | --- | --- | --- | --- | --- |
| H7 | 4.623645 | B36 | 4.733237 | A44 | 7.118685 | F5 | 5.766121 | B12 | 6.416546 |
| F11 | 5.203448 | B38 | 5.654848 | A45 | 4.870504 | E14 | 5.203383 | A2 | 5.722862 |
| F12 | 6.48875 | B40 | 4.945365 | A46 | 5.292285 | F9 | 5.600408 | A3 | 6.185439 |
| E16 | 4.802887 | A48 | 5.60382 | A47 | 5.157559 | F10 | 5.103687 | A4 | 4.683875 |
| M15 | 4.450454 | B43 | 5.876138 | A49 | 5.55288 | E15 | 6.415448 | A5 | 4.755608 |
| M16 | 5.413696 | M6 | 6.143486 | A50 | 4.983078 | K4 | 5.965769 | A8 | 4.461045 |
| H21 | 6.070221 | N1 | 5.875419 | A51 | 4.45053 | K6 | 4.596912 | B13 | 5.307784 |
| E10 | 5.478258 | F3 | 5.688143 | A52 | 5.680373 | H14 | 6.591526 | E3 | 5.613014 |
| K5 | 5.644852 | H10 | 5.365925 | B42 | 5.212843 | H15 | 5.571256 | A9 | 6.708361 |
| H19 | 4.998444 | E8 | 4.83275 | M2 | 5.780109 | K7 | 6.135322 | A10 | 5.823795 |
| F14 | 5.25844 | N2 | 5.709385 | B44 | 4.983845 | H4 | 5.569968 | B14 | 4.732929 |
| E27 | 4.415747 | H1 | 6.834535 | B45 | 5.441186 | H16 | 5.697288 | A11 | 6.120944 |
| H13 | 5.88415 | E5 | 4.641125 | B46 | 6.204133 | H17 | 5.436605 | B15 | 4.669089 |
| E11 | 5.276292 | B22 | 5.769616 | A53 | 5.678685 | E17 | 4.227471 | A12 | 5.179051 |
| F6 | 4.902329 | B23 | 6.263862 | B47 | 4.814455 | H18 | 5.406295 | A13 | 5.354039 |
| E13 | 4.942408 | A26 | 5.628038 | B48 | 6.225336 | E18 | 5.047883 | B16 | 6.779198 |
| F7 | 5.260641 | B24 | 5.568941 | B49 | 6.158736 | M11 | 4.278143 | B17 | 5.828363 |
| F8 | 5.462931 | B25 | 4.643987 | C1 | 5.235263 | M13 | 5.021387 | A15 | 5.840087 |
| K2 | 6.088173 | B26 | 7.201781 | C2 | 5.104441 | M14 | 5.227346 | E4 | 6.458642 |
| K3 | 5.337429 | B27 | 6.493621 | C3 | 6.050544 | N3 | 5.885519 | A16 | 6.262371 |
| H3 | 6.940199 | A29 | 3.911045 | M3 | 4.862538 | M17 | 5.669306 | A17 | 5.133624 |
| M12 | 5.066009 | A30 | 7.640589 | C4 | 5.103964 | H5 | 5.759528 | A18 | 7.536004 |
| M20 | 5.39608 | A32 | 5.295539 | C5 | 6.201141 | M18 | 6.056334 | A19 | 5.074044 |
| M21 | 5.182844 | H8 | 5.100434 | C6 | 6.316562 | M19 | 4.292968 | A20 | 6.991567 |
| M22 | 4.306681 | A33 | 6.541464 | C7 | 5.098176 | M24 | 5.454881 | A21 | 5.600695 |
| M23 | 5.594417 | A35 | 4.878368 | C8 | 5.640436 | M25 | 4.979165 | B19 | 5.802101 |
| F15 | 5.271441 | A36 | 5.133374 | C9 | 4.946538 | F13 | 5.273404 | B20 | 4.381184 |
| H20 | 7.107245 | A37 | 5.097296 | C10 | 4.952245 | E19 | 4.819136 | A22 | 7.579774 |
| E22 | 5.049233 | A38 | 6.001598 | C11 | 5.654189 | K8 | 4.76489 | A24 | 5.542705 |
| E23 | 4.411831 | A39 | 4.667999 | C12 | 4.970618 | E20 | 5.006447 | H23 | 6.963903 |
| E26 | 6.734976 | A40 | 6.791901 | C13 | 5.113379 | E21 | 5.137806 | G6 | 5.412141 |
| H9 | 6.042523 | A41 | 6.579487 | M4 | 4.869274 | E24 | 5.185934 | G7 | 7.586182 |
| B1 | 4.889214 | B28 | 5.667872 | M5 | 5.348366 | E2 | 5.114997 | G8 | 6.126548 |
| B2 | 5.077595 | A42 | 6.05784 | K1 | 6.708933 | E25 | 5.103514 | G9 | 6.162383 |
| B4 | 6.610285 | E6 | 5.143142 | M7 | 5.431192 | E28 | 5.487684 | M26 | 5.236139 |
| A6 | 4.354981 | A43 | 4.821576 | H2 | 6.544559 | H22 | 5.474183 | M27 | 4.728972 |
| A7 | 6.570985 | B29 | 4.786275 | M8 | 4.920711 | K9 | 5.036893 | M28 | 4.486799 |
| A14 | 5.827249 | B30 | 5.695486 | M9 | 5.803027 | B3 | 4.641659 | F16 | 5.844282 |
| B18 | 5.093054 | B31 | 6.160242 | M10 | 5.111475 | B5 | 6.160432 | K10 | 4.792686 |
| B21 | 5.227333 | B32 | 6.418585 | F1 | 6.256365 | B6 | 6.040195 | G1 | 4.740977 |
| A23 | 4.71022 | B33 | 5.486512 | F2 | 4.811413 | B7 | 5.200376 | K11 | 4.130735 |
| A25 | 5.336917 | B34 | 4.967323 | F4 | 5.302897 | B8 | 5.630274 | G2 | 5.730283 |
| M1 | 5.727091 | B35 | 4.860708 | H11 | 6.234144 | A1 | 5.034698 | G3 | 5.965548 |
| A27 | 5.862258 | B37 | 6.027077 | H12 | 6.80692 | H6 | 5.631119 | G4 | 5.736121 |
| A28 | 4.702633 | B39 | 6.211613 | E9 | 5.168935 | B9 | 6.1222 | G5 | 5.175402 |
| A31 | 6.321039 | E7 | 5.267607 | E12 | 4.779442 | B10 | 5.949377 |  |  |
| A34 | 7.350722 | B41 | 5.943644 | E1 | 7.259163 | B11 | 5.883135 |  |  |

**Supplementary Tables 5.** Q-value statistics.

| **ID** | **subpopulation I** | **subpopulation II** | **subpopulation III** | **subpopulation IV** | **ID** | **subpopulation I** | **subpopulation II** | **subpopulation III** | **subpopulation IV** |
| --- | --- | --- | --- | --- | --- | --- | --- | --- | --- |
| H7 | 0.00001 | 0.99997 | 0.00001 | 0.00001 | B41 | 0.382056 | 0.146087 | 0.137462 | 0.334395 |
| C1 | 0.401792 | 0.211512 | 0.163626 | 0.22307 | A44 | 0.443914 | 0.176709 | 0.154509 | 0.224868 |
| C2 | 0.380289 | 0.21736 | 0.147645 | 0.254707 | A45 | 0.597159 | 0.120704 | 0.154405 | 0.127731 |
| C3 | 0.425382 | 0.189194 | 0.162621 | 0.222803 | A46 | 0.672858 | 0.077774 | 0.097124 | 0.152243 |
| C4 | 0.426291 | 0.181589 | 0.157069 | 0.235051 | M2 | 0.495087 | 0.089251 | 0.195341 | 0.220321 |
| C5 | 0.371862 | 0.188739 | 0.172035 | 0.267364 | B44 | 0.523357 | 0.139098 | 0.138125 | 0.19942 |
| C6 | 0.38828 | 0.194185 | 0.179474 | 0.238061 | B45 | 0.504433 | 0.139278 | 0.106764 | 0.249525 |
| E10 | 0.051721 | 0.027457 | 0.058726 | 0.862095 | B46 | 0.394864 | 0.166501 | 0.152611 | 0.286024 |
| C7 | 0.394232 | 0.201429 | 0.176373 | 0.227966 | B47 | 0.554217 | 0.158484 | 0.111665 | 0.175633 |
| C8 | 0.35908 | 0.204084 | 0.18446 | 0.252376 | H20 | 0.287773 | 0.413419 | 0.136676 | 0.162131 |
| K3 | 0.00001 | 0.869848 | 0.00001 | 0.130132 | A49 | 0.155445 | 0.087462 | 0.642176 | 0.114917 |
| C9 | 0.408961 | 0.193759 | 0.140824 | 0.256456 | F6 | 0.00001 | 0.008425 | 0.00001 | 0.991555 |
| C10 | 0.38981 | 0.225365 | 0.16912 | 0.215705 | E26 | 0.062119 | 0.714469 | 0.051251 | 0.172161 |
| C11 | 0.416908 | 0.190519 | 0.161297 | 0.231276 | A34 | 0.182199 | 0.714471 | 0.050606 | 0.052723 |
| M1 | 0.297953 | 0.147854 | 0.153165 | 0.401028 | M8 | 0.217157 | 0.393293 | 0.38954 | 0.00001 |
| B42 | 0.283661 | 0.30549 | 0.086826 | 0.324023 | M9 | 0.047581 | 0.492141 | 0.334256 | 0.126022 |
| M7 | 0.266422 | 0.211932 | 0.253686 | 0.26796 | B48 | 0.656869 | 0.1133 | 0.029631 | 0.200199 |
| M14 | 0.315789 | 0.210137 | 0.123328 | 0.350746 | B49 | 0.649934 | 0.113223 | 0.032788 | 0.204055 |
| M16 | 0.434076 | 0.164563 | 0.178518 | 0.222844 | M3 | 0.852366 | 0.00001 | 0.120677 | 0.026947 |
| H9 | 0.257583 | 0.419746 | 0.155367 | 0.167304 | A51 | 0.097549 | 0.123909 | 0.710172 | 0.06837 |
| M20 | 0.37327 | 0.181294 | 0.159157 | 0.286279 | M10 | 0.015339 | 0.514026 | 0.315649 | 0.154986 |
| M23 | 0.461331 | 0.202818 | 0.191156 | 0.144695 | A52 | 0.076978 | 0.131543 | 0.710977 | 0.080502 |
| H8 | 0.380545 | 0.226974 | 0.155385 | 0.237096 | E13 | 0.00001 | 0.00001 | 0.00001 | 0.99997 |
| A35 | 0.356194 | 0.169837 | 0.18182 | 0.292149 | F7 | 0.00001 | 0.00001 | 0.00001 | 0.99997 |
| H10 | 0.120237 | 0.745975 | 0.072593 | 0.061195 | F8 | 0.098967 | 0.049064 | 0.00001 | 0.85196 |
| A36 | 0.678289 | 0.156528 | 0.034662 | 0.13052 | F1 | 0.631746 | 0.11363 | 0.08162 | 0.173005 |
| M6 | 0.078391 | 0.00001 | 0.910633 | 0.010966 | M19 | 0.00001 | 0.927667 | 0.00001 | 0.072313 |
| E5 | 0.00001 | 0.99997 | 0.00001 | 0.00001 | E12 | 0.00001 | 0.00001 | 0.00001 | 0.99997 |
| A37 | 0.437503 | 0.18289 | 0.13304 | 0.246567 | E15 | 0.403729 | 0.185239 | 0.182887 | 0.228145 |
| B32 | 0.477548 | 0.033496 | 0.413306 | 0.075651 | E20 | 0.306148 | 0.384404 | 0.14053 | 0.168918 |
| M5 | 0.012781 | 0.761106 | 0.142365 | 0.083747 | E1 | 0.167295 | 0.00001 | 0.138017 | 0.694678 |
| K4 | 0.00001 | 0.873471 | 0.00001 | 0.126509 | F5 | 0.00001 | 0.00001 | 0.00001 | 0.99997 |
| B34 | 0.55372 | 0.119716 | 0.130352 | 0.196213 | F10 | 0.24371 | 0.143155 | 0.12248 | 0.490655 |
| A50 | 0.498965 | 0.065207 | 0.169189 | 0.266638 | K1 | 0.00001 | 0.029267 | 0.910082 | 0.060641 |
| A53 | 0.448205 | 0.106969 | 0.241152 | 0.203673 | H4 | 0.468371 | 0.157828 | 0.146925 | 0.226875 |
| C12 | 0.379993 | 0.21764 | 0.16681 | 0.235557 | E17 | 0.731025 | 0.00001 | 0.192753 | 0.076212 |
| N1 | 0.080693 | 0.00001 | 0.908328 | 0.010969 | H11 | 0.00001 | 0.030605 | 0.957551 | 0.011834 |
| C13 | 0.419235 | 0.21415 | 0.15678 | 0.209835 | N3 | 0.333244 | 0.184391 | 0.247392 | 0.234973 |
| M4 | 0.428096 | 0.178343 | 0.148859 | 0.244702 | M24 | 0.597309 | 0.004215 | 0.315455 | 0.083021 |
| F2 | 0.392165 | 0.189742 | 0.200135 | 0.217958 | F13 | 0.301363 | 0.188554 | 0.263166 | 0.246917 |
| F4 | 0.463915 | 0.145659 | 0.107407 | 0.283019 | K6 | 0.00001 | 0.00001 | 0.00001 | 0.99997 |
| H16 | 0.326627 | 0.144442 | 0.265314 | 0.263616 | E19 | 0.642325 | 0.035708 | 0.23267 | 0.089298 |
| B3 | 0.322888 | 0.196152 | 0.121862 | 0.359098 | H14 | 0.138663 | 0.011181 | 0.085139 | 0.765017 |
| H18 | 0.536421 | 0.094967 | 0.196703 | 0.17191 | E24 | 0.743208 | 0.038753 | 0.127784 | 0.090256 |
| E18 | 0.334958 | 0.316495 | 0.064953 | 0.283594 | E9 | 0.089101 | 0.00001 | 0.90669 | 0.004199 |
| M18 | 0.334833 | 0.177035 | 0.274112 | 0.214021 | H15 | 0.21708 | 0.00001 | 0.052772 | 0.730138 |
| E4 | 0.00001 | 0.99997 | 0.00001 | 0.00001 | H22 | 0.446212 | 0.174555 | 0.117892 | 0.261341 |
| H6 | 0.396387 | 0.197458 | 0.163675 | 0.24248 | E2 | 0.124319 | 0.772361 | 0.036284 | 0.067036 |
| M17 | 0.10179 | 0.022334 | 0.867599 | 0.008277 | K9 | 0.401955 | 0.180105 | 0.116273 | 0.301667 |
| B13 | 0.416316 | 0.192664 | 0.18938 | 0.20164 | B5 | 0.416531 | 0.18345 | 0.165225 | 0.234795 |
| A15 | 0.769408 | 0.03854 | 0.065568 | 0.126484 | B6 | 0.405675 | 0.225451 | 0.190424 | 0.17845 |
| A21 | 0.42684 | 0.206478 | 0.121629 | 0.245053 | B7 | 0.747562 | 0.068527 | 0.079655 | 0.104255 |
| K8 | 0.09631 | 0.024935 | 0.878745 | 0.00001 | H17 | 0.156666 | 0.00001 | 0.83732 | 0.006004 |
| G3 | 0.069427 | 0.054002 | 0.866536 | 0.010035 | H2 | 0.179718 | 0.497641 | 0.102736 | 0.219906 |
| F15 | 0.23132 | 0.00001 | 0.76866 | 0.00001 | B8 | 0.575091 | 0.102171 | 0.122171 | 0.200568 |
| F14 | 0.335099 | 0.655782 | 0.000977 | 0.008143 | A1 | 0.582997 | 0.121266 | 0.108703 | 0.187035 |
| G9 | 0.409646 | 0.194888 | 0.154629 | 0.240837 | H12 | 0.297525 | 0.410176 | 0.12844 | 0.163859 |
| F11 | 0.415328 | 0.189616 | 0.190742 | 0.204314 | M11 | 0.0701 | 0.030335 | 0.063174 | 0.836391 |
| F12 | 0.442421 | 0.18156 | 0.183077 | 0.192942 | B9 | 0.484465 | 0.166987 | 0.138423 | 0.210126 |
| M22 | 0.00001 | 0.99997 | 0.00001 | 0.00001 | B10 | 0.396749 | 0.159357 | 0.157298 | 0.286596 |
| M15 | 0.514264 | 0.158883 | 0.143756 | 0.183097 | B11 | 0.438056 | 0.17548 | 0.147487 | 0.238977 |
| H21 | 0.323415 | 0.195959 | 0.204764 | 0.275861 | H5 | 0.308841 | 0.20237 | 0.16543 | 0.323358 |
| H19 | 0.414631 | 0.142925 | 0.180211 | 0.262233 | B12 | 0.840322 | 0.066446 | 0.022487 | 0.070745 |
| E27 | 0.379783 | 0.169688 | 0.20568 | 0.24485 | A2 | 0.52463 | 0.136377 | 0.117203 | 0.22179 |
| M21 | 0.601232 | 0.00001 | 0.318856 | 0.079902 | A3 | 0.855925 | 0.069678 | 0.002525 | 0.071873 |
| E23 | 0.604492 | 0.096222 | 0.155999 | 0.143288 | A4 | 0.657294 | 0.088194 | 0.113608 | 0.140905 |
| B1 | 0.470343 | 0.130786 | 0.156966 | 0.241905 | A5 | 0.400816 | 0.201831 | 0.161983 | 0.23537 |
| B2 | 0.746704 | 0.116643 | 0.021156 | 0.115497 | A8 | 0.456259 | 0.169029 | 0.145963 | 0.228749 |
| B4 | 0.44423 | 0.152851 | 0.184509 | 0.21841 | A9 | 0.445589 | 0.189634 | 0.166516 | 0.198261 |
| A6 | 0.493188 | 0.17369 | 0.132576 | 0.200546 | A10 | 0.568124 | 0.086991 | 0.141831 | 0.203054 |
| A14 | 0.560099 | 0.031116 | 0.320351 | 0.088434 | B14 | 0.469893 | 0.189812 | 0.159579 | 0.180717 |
| B18 | 0.484759 | 0.101757 | 0.230783 | 0.1827 | B15 | 0.702573 | 0.05704 | 0.079213 | 0.161174 |
| B21 | 0.451599 | 0.212001 | 0.154856 | 0.181544 | A13 | 0.496369 | 0.153867 | 0.166718 | 0.183045 |
| A23 | 0.556804 | 0.074113 | 0.269687 | 0.099396 | B16 | 0.422145 | 0.091052 | 0.373409 | 0.113395 |
| A25 | 0.615525 | 0.018075 | 0.278024 | 0.088376 | B17 | 0.910676 | 0.034269 | 0.00001 | 0.055045 |
| A27 | 0.631081 | 0.030025 | 0.281296 | 0.057599 | A16 | 0.585148 | 0.09677 | 0.11713 | 0.200952 |
| A28 | 0.51004 | 0.005344 | 0.324137 | 0.160479 | A17 | 0.564643 | 0.076486 | 0.174583 | 0.184287 |
| A31 | 0.74316 | 0.021352 | 0.14753 | 0.087957 | A18 | 0.615768 | 0.117523 | 0.105781 | 0.160928 |
| B36 | 0.916507 | 0.038544 | 0.00001 | 0.044939 | E3 | 0.00001 | 0.038234 | 0.897581 | 0.064176 |
| B40 | 0.425377 | 0.138432 | 0.218186 | 0.218005 | A19 | 0.433573 | 0.137934 | 0.162889 | 0.265605 |
| A48 | 0.703983 | 0.03884 | 0.121499 | 0.135679 | A20 | 0.503519 | 0.1347 | 0.130561 | 0.23122 |
| B43 | 0.522435 | 0.00001 | 0.413604 | 0.06395 | B19 | 0.503161 | 0.14248 | 0.126686 | 0.227673 |
| E8 | 0.457713 | 0.191832 | 0.12071 | 0.229745 | A11 | 0.231858 | 0.168017 | 0.405898 | 0.194227 |
| N2 | 0.405289 | 0.167505 | 0.116424 | 0.310782 | B20 | 0.785187 | 0.019792 | 0.195011 | 0.00001 |
| H1 | 0.378238 | 0.216264 | 0.134334 | 0.271164 | A12 | 0.145647 | 0.085422 | 0.651014 | 0.117917 |
| B22 | 0.611964 | 0.025171 | 0.141256 | 0.221609 | A22 | 0.600358 | 0.026497 | 0.209172 | 0.163972 |
| B23 | 0.438477 | 0.172457 | 0.147314 | 0.241752 | A24 | 0.560586 | 0.111897 | 0.127827 | 0.19969 |
| A26 | 0.750865 | 0.044514 | 0.110255 | 0.094366 | K10 | 0.472718 | 0.117716 | 0.134463 | 0.275103 |
| B24 | 0.453269 | 0.170226 | 0.140022 | 0.236482 | E16 | 0.329407 | 0.198358 | 0.157037 | 0.315199 |
| A7 | 0.079557 | 0.036206 | 0.871811 | 0.012425 | F9 | 0.056802 | 0.683881 | 0.035483 | 0.223833 |
| B25 | 0.720204 | 0.108398 | 0.033584 | 0.137815 | K5 | 0.451343 | 0.169619 | 0.155958 | 0.22308 |
| B26 | 0.657423 | 0.082216 | 0.129499 | 0.130862 | H13 | 0.39591 | 0.148366 | 0.179516 | 0.276208 |
| B38 | 0.152165 | 0.08573 | 0.636904 | 0.125201 | E11 | 0.513696 | 0.144619 | 0.084944 | 0.256742 |
| B27 | 0.651596 | 0.05726 | 0.164486 | 0.126658 | K2 | 0.345629 | 0.212973 | 0.137999 | 0.303399 |
| A29 | 0.39373 | 0.179448 | 0.162254 | 0.264568 | H3 | 0.38563 | 0.178903 | 0.16814 | 0.267328 |
| A30 | 0.85659 | 0.049308 | 0.024121 | 0.069981 | M12 | 0.853214 | 0.00001 | 0.119902 | 0.026873 |
| A32 | 0.483718 | 0.188243 | 0.117725 | 0.210314 | E22 | 0.656548 | 0.000346 | 0.236015 | 0.107091 |
| F3 | 0.00001 | 0.036207 | 0.931553 | 0.03223 | E14 | 0.405874 | 0.195193 | 0.129239 | 0.269694 |
| B35 | 0.303243 | 0.160198 | 0.380989 | 0.15557 | M13 | 0.388293 | 0.164826 | 0.192975 | 0.253906 |
| A33 | 0.481631 | 0.112236 | 0.318952 | 0.087181 | M25 | 0.391883 | 0.227786 | 0.139839 | 0.240492 |
| E7 | 0.105629 | 0.00001 | 0.861981 | 0.032379 | E25 | 0.271539 | 0.183417 | 0.122805 | 0.422238 |
| A47 | 0.165149 | 0.109897 | 0.626235 | 0.098719 | E21 | 0.476608 | 0.091997 | 0.146621 | 0.284774 |
| F16 | 0.332611 | 0.207206 | 0.125758 | 0.334425 | E28 | 0.418857 | 0.113628 | 0.154308 | 0.313207 |
| A38 | 0.544504 | 0.088177 | 0.089823 | 0.277496 | H23 | 0.259508 | 0.156733 | 0.16654 | 0.41722 |
| A39 | 0.521725 | 0.081937 | 0.298809 | 0.09753 | G6 | 0.414166 | 0.193278 | 0.1535 | 0.239055 |
| A40 | 0.819332 | 0.092095 | 0.00001 | 0.088563 | G8 | 0.068279 | 0.113514 | 0.044772 | 0.773435 |
| A41 | 0.562938 | 0.036016 | 0.018336 | 0.38271 | G7 | 0.401168 | 0.194242 | 0.140666 | 0.263924 |
| B28 | 0.58923 | 0.102635 | 0.094706 | 0.213429 | M27 | 0.381791 | 0.190988 | 0.132348 | 0.294873 |
| A42 | 0.789139 | 0.00001 | 0.138323 | 0.072528 | M26 | 0.320197 | 0.139083 | 0.126155 | 0.414566 |
| E6 | 0.400822 | 0.144043 | 0.198414 | 0.256721 | M28 | 0.353154 | 0.175549 | 0.139871 | 0.331425 |
| A43 | 0.61241 | 0.081615 | 0.125262 | 0.180713 | G1 | 0.097227 | 0.01352 | 0.0821 | 0.807154 |
| B29 | 0.426378 | 0.206958 | 0.151655 | 0.215009 | K11 | 0.390304 | 0.183266 | 0.132583 | 0.293847 |
| B30 | 0.595154 | 0.080289 | 0.095169 | 0.229388 | G2 | 0.098495 | 0.759863 | 0.036209 | 0.105433 |
| B31 | 0.395061 | 0.213841 | 0.143435 | 0.247663 | K7 | 0.16693 | 0.093837 | 0.612014 | 0.127219 |
| B33 | 0.702762 | 0.00001 | 0.255283 | 0.041944 | G4 | 0.327357 | 0.206877 | 0.135269 | 0.330496 |
| B37 | 0.547022 | 0.12232 | 0.134595 | 0.196063 | G5 | 0.255689 | 0.169891 | 0.152479 | 0.421941 |
| B39 | 0.557542 | 0.072822 | 0.253726 | 0.115909 |  |  |  |  |  |
